# Glucagon reduces airway hyperreactivity, inflammation, and remodeling induced by ovalbumin

**DOI:** 10.1038/s41598-019-42981-6

**Published:** 2019-04-24

**Authors:** Daniella B. R. Insuela, Carolina T. Azevedo, Diego S. Coutinho, Nathalia S. Magalhães, Maximiliano R. Ferrero, Tatiana Paula T. Ferreira, Cynthia M. Cascabulho, Andrea Henriques-Pons, Priscilla C. Olsen, Bruno L. Diaz, Patricia M. R. Silva, Renato S. B. Cordeiro, Marco A. Martins, Vinicius F. Carvalho

**Affiliations:** 1 Laboratory of Inflammation, Oswaldo Cruz Institute, Oswaldo Cruz Foundation (FIOCRUZ), Rio de Janeiro, Brazil; 2 Laboratory of Innovations in Therapies, Education and Bioproducts, Oswaldo Cruz Institute, Oswaldo Cruz Foundation (FIOCRUZ), Rio de Janeiro, Brazil; 3 Laboratory of Clinical Bacteriology and Immunology, Department of Toxicological and Clinical Analysis, Faculty of Pharmacy, Federal University of Rio de Janeiro, Rio de Janeiro, Brazil; 4 Laboratory of Inflammation, Carlos Chagas Filho Institute of Biophysics, Federal University of Rio de Janeiro, Rio de Janeiro, Brazil; 5 National Institute of Science and Technology on Neuroimmunomodulation (INCT-NIM), Rio de Janeiro, Brazil

**Keywords:** Chronic inflammation, Respiration

## Abstract

Glucagon has been shown to be beneficial as a treatment for bronchospasm in asthmatics. Here, we investigate if glucagon would prevent airway hyperreactivity (AHR), lung inflammation, and remodeling in a murine model of asthma. Glucagon (10 and 100 µg/Kg, i.n.) significantly prevented AHR and eosinophilia in BAL and peribronchiolar region induced by ovalbumin (OVA) challenge, while only the dose of 100 µg/Kg of glucagon inhibited subepithelial fibrosis and T lymphocytes accumulation in BAL and lung. The inhibitory action of glucagon occurred in parallel with reduction of OVA-induced generation of IL-4, IL-5, IL-13, TNF-α, eotaxin-1/CCL11, and eotaxin-2/CCL24 but not MDC/CCL22 and TARC/CCL17. The inhibitory effect of glucagon (100 µg/Kg, i.n.) on OVA-induced AHR and collagen deposition was reversed by pre-treatment with indomethacin (10 mg/Kg, i.p.). Glucagon increased intracellular cAMP levels and inhibits anti-CD3 plus anti-CD28-induced proliferation and production of IL-2, IL-4, IL-10, and TNF- α from TCD4^+^ cells *in vitro*. These findings suggest that glucagon reduces crucial features of asthma, including AHR, lung inflammation, and remodeling, in a mechanism probably associated with inhibition of eosinophils accumulation and TCD4^+^ cell proliferation and function. Glucagon should be further investigated as an option for asthma therapy.

## Introduction

Asthma is a chronic lung disease characterized by persistent inflammation in association with airway hyperreactivity (AHR) and tissue remodeling^1^. It is considered the 14^th^ most important disorder in the world, affecting more than 334 million people globally^2^. The main inflammatory cells observed in asthmatic lungs are mast cells, eosinophils, and TCD4^+^ effector lymphocytes^3^. In regards to T lymphocytes, Th2 cells are considered one of the central players in asthma pathogenesis, as long as these cells participate in and coordinate the onset and progression of the inflammatory response in asthma through the release of cytokines, especially IL-4, IL-5, IL-9, and IL-13. Indeed, the levels of these cytokines are increased in asthmatic patients^4^.

Currently, the combination of β_2_ agonists with corticosteroids represents the gold standard therapy for asthma. However, a significant number of patients does not respond to corticosteroid treatment, even when high doses of corticosteroids are used^5^. Therefore, the search for new and broadly effective asthma treatments is still necessary, especially treatments that have both bronchodilator and anti-inflammatory effects^6^.

Glucagon is a hormone secreted by α-pancreatic cells in response to low glucose levels or high concentrations of amino acids^7^. The main physiological function of glucagon is to maintain glucose homeostasis in cases of hypoglycemia^7,8^. However, glucagon has alternative actions, which may show much promise for clinical application, such as its effect on respiratory smooth muscle contraction and the inflammatory response^9^. Prior investigations demonstrate that glucagon causes bronchial relaxation in patients with asthma, including those suffering from refractory asthma exacerbation^10–12^. Furthermore, glucagon inhibits mast cell number and activation^13^ as well as LPS-induced AHR and accumulation of neutrophils in the bronchoalveolar fluid^14^.

Until now, it has been known that glucagon has bronchodilator effects both in animal models^14,15^ and in asthmatic patients^10^, however little is known about the anti-inflammatory effects of this hormone in asthma. The purpose of this study was to evaluate the effectiveness of intranasal glucagon on the development of the airway inflammation and tissue remodeling in a murine model of asthma. The putative mechanistic implication of T cells in this effect was also explored. The novelty of this study is that we showed that glucagon has an anti-inflammatory effect in a model of allergen-induced lung inflammation, by inhibiting AHR, inflammatory response, and remodeling, in a mechanism related to decrease of eosinophils and T cell accumulation.

## Results

### Mice challenged with ovalbumin present an increased glucagon receptor expression in bronchoalveolar lavage cells, mediastinal lymph nodes and lungs

**Figure 1:**
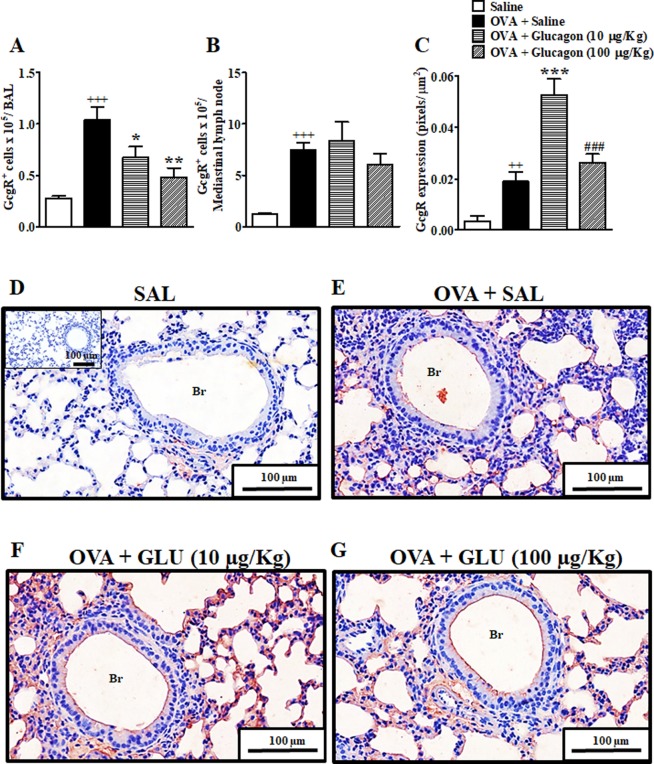
Glucagon inhibits the increased number of GcgR^+^ cells in BAL and rise the expression of GcgR in lungs induced by OVA. The animals were challenged i.n. with OVA (25 μg/25 μL) or sterile saline (0.9%) once a day for 2 consecutive days and the treatment with glucagon (10 and 100 µg/Kg, i.n.) or sterile saline (0.9%) was performed 1 h before each challenge with OVA. BAL was collected, and mediastinal lymph node and lungs were removed for analysis 24 h after the last challenge. The number of cells expressing GcgR in BAL (**A**) and mediastinal lymph node (**B**) of A/J mice after i.n. challenged with OVA and treated with saline. Quantification of pixels corresponding to positive GcgR expression in lungs (**C**). Representative photomicrographs of GcgR expression on bronchioles and peribronchiolar region of lungs from saline-challenged (0.9%, i.n.) (**D**), OVA-challenged treated with saline (**E**) and OVA-challenged mice treated with glucagon 10 (**F**) or 100 µg/Kg, i.n. (**G**). The results are expressed as the mean ± SEM of 3–6 animals per group. Statistical analysis was performed using a one-way ANOVA followed by Newman–Keuls-Student’s T test. ^++^*P* < 0.01 compared to the group challenged with saline. ^+++^*P* < 0.001 compared to the group challenged with saline. *P < 0.05 compared to the group challenge with OVA plus saline. **P < 0.01 compared to the group challenge with OVA plus saline. ***P < 0.001 compared to the group challenge with OVA plus saline. ^###^*P* < 0.001 compared to the group challenge with OVA and treated with glucagon 10 µg/Kg. Br = Bronchiolar lumen. SAL = Saline. OVA = Ovalbumin. GLU = Glucagon.

Sensitized mice challenged with ovalbumin (OVA) showed an increase in the number of cells that express glucagon receptor (GcgR) in bronchoalveolar lavage (BAL) (Fig. 1A), including Total CD3^+^ cells (T cells), TCD4^+^ cells, dendritic cells, and neutrophils (Fig. S1F), and mediastinal lymph node (Fig. 1B), when compared to mice challenged with sterile saline. However, we did not noted alterations in the numbers of eosinophils that express GcgR in BAL (Fig. S1F). Furthermore, we observed an increase of GcgR expression in lung tissue samples of mice submitted to OVA challenge (Fig. 1C,E) when compared with lung samples from control mice challenged with sterile saline (Fig. 1C,D). We noted that the increase in immunolabeling for GcgR in lungs from OVA-challenged mice occurs preferentially in inflammatory cells (Fig. 1E). We showed that this increase in the expression of GcgR receptor was detected in both mononuclear and polymorphonuclear cells, but not in airway epithelial cells and airway smooth muscle cells (Fig. S1). In addition, intranasal instillation of glucagon at 10 and 100 µg/Kg reduced the numbers of cells which express GcgR in BAL (Fig. 1A), but not in mediastinal lymph node (Fig. 1B). Furthermore, glucagon at 10 µg/Kg (Fig. 1C,F), but not at 100 µg/Kg (Fig. 1C,G), increased the GcgR expression in lung tissue samples of mice submitted to OVA challenge.

### Glucagon prevents AHR to methacholine in A/J mice challenged with OVA

**Figure 2:**
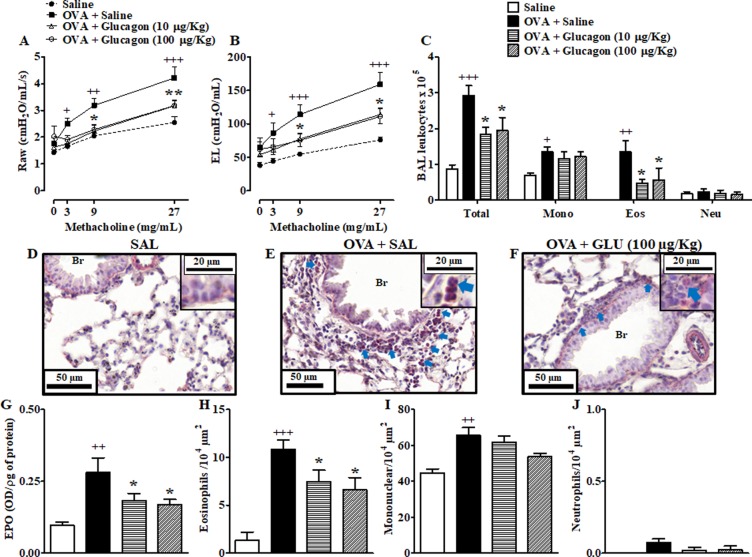
Glucagon prevents OVA-induced AHR to methacholine, and infiltration of eosinophils in BAL and lung of A/J mice. The treatment with glucagon (10 and 100 µg/Kg, i.n.) or its vehicle (sterile saline 0.9%, i.n.) was performed 1 h before each challenge with OVA, once a day for 2 consecutive days. Glucagon inhibited the elevation on Raw (**A**) and EL (**B**) induced by increasing concentrations of methacholine (3–27 mg/mL) in A/J mice challenged i.n. with sterile saline (0.9%) or OVA (25 μg/25 μL). The results are expressed as the mean ± SEM of 7–8 animals per group. Statistical analysis was performed using a two-way ANOVA followed by Bonferroni post-test. Glucagon inhibited total leukocyte and eosinophil accumulation in BAL (**C**) and EPO activity in lungs (**G**), 24 h after the last challenge with OVA (25 μg/25 μL) or saline. Photomicrographs of representative airways in Sirius Red-stained lung sections from mice challenged with saline (**D**), OVA plus saline (**E**), and OVA plus glucagon (100 μg/Kg) (**F**). Blue arrows indicate eosinophils. The number of eosinophils (**H**), mononuclear cells (**I**) and neutrophils (**J**) in peribronchiolar regions were counted in 7–10 bronchioles per mouse. The results are expressed as the mean ± SEM of 5 animals per group to inflammatory cells accumulation in the BAL; 8–10 animals per group to EPO activity; and 3–5 animals per group to histological analyses. Statistical analysis was performed using a one-way ANOVA followed by Newman–Keuls-Student’s T test. ^+^*P* < 0.05 compared to the group challenged with saline. ^++^*P* < 0.01 compared to the group challenged with saline. ^+++^*P* < 0.001 compared to the group challenged with saline. *P < 0.05 compared to the group challenged with OVA plus saline. **P < 0.01 compared to the group challenged with OVA OVA plus saline. Br = Bronchiolar lumen. Eos = Eosinophils. Mono = Mononuclear. Neu = Neutrophils. SAL = Saline. OVA = Ovalbumin. GLU = Glucagon.

As expected, exposure of mice to aerosolized methacholine increased airway resistance (Raw) (Fig. 2A) and lung elastance (EL) (Fig. 2B) in a concentration-dependent manner. Compared with sham-challenged mice, these responses were significantly higher in those mice sensitized and challenged with OVA, characterizing the state of allergen-induced AHR. Our findings revealed that intranasal instillation of 10 or 100 μg/Kg glucagon, 1 h before allergen challenge, equally inhibited AHR concerning Raw and EL (Fig. 2A,B); however treatment with glucagon at doses of 0.1 and 1 µg/Kg did not decrease OVA-induced AHR (Fig. S4A and S4B).

### Glucagon treatment prevents lung inflammation induced by OVA

OVA-challenged mice showed an increase in total leukocyte numbers detected in BAL, when compared to sensitized mice challenged with sterile saline. This increase in cell counts was accounted for by elevations in numbers of mononuclear cells and eosinophils without changes in neutrophil counts (Fig. 2C). As shown in Fig. 2C, treatment with either 10 or 100 µg/Kg of glucagon, 1 h before allergen challenge significantly inhibited eosinophil but not mononuclear cell accumulation in BAL effluent, but at 0.1 and 1 µg/Kg glucagon was unable to alter the infiltration of these cells (Fig. S4C). Next, we used the eosinophil peroxidase (EPO) activity assay method to indirectly quantify the presence of eosinophils in lung tissue since there is a direct relationship between eosinophil accumulation and the measurement of EPO activity in lung tissue extracts^14^. In OVA-challenged mice, there was an increase in EPO activity in lung tissue extract, compared with sham-challenged mice, which were sensitive to glucagon (Fig. 2G).

We also performed histological analyses of the lung tissue to quantify peribronchiolar eosinophil, neutrophil, and mononuclear cell numbers after challenge with OVA or saline. Mice challenged with OVA presented mononuclear and eosinophilic but not neutrophilic infiltration, compared with mice challenged with saline. The histologic analysis of the leukocyte infiltrate revealed that glucagon at 10 and 100 µg/kg equally inhibited the peribronchiolar eosinophilic response without significantly interfering with mononuclear cells and neutrophil changes despite the trend. Representative photomicrographs are shown in Fig. 2D–F for saline, OVA plus saline, and OVA plus glucagon (100 µg/Kg, i.n.) groups, respectively, whereas quantitative data are shown in Fig. 2H–J for measurement of eosinophils, mononuclear cells and neutrophils, respectively.

### Glucagon treatment prevents the OVA-induced lung accumulation of Tαβ lymphocytes

Due to the importance of T lymphocytes in the pathophysiology of asthma, we evaluated the effect of glucagon upon accumulation of Tαβ lymphocytes in BAL, mediastinal lymph nodes and the lungs of mice challenged with OVA. In our experiment, both TCD4^+^ and TCD8^+^ cells obtained from BAL express GcgR. The percentage of cells expressing GcgR was 41.1 ± 8.3% (n = 5) and 21.2 ± 3.4% (n = 4) (mean ± standard deviation) for TCD4^+^ and TCD8^+^ cells, respectively, in BAL fluid. In mediastinal lymph nodes we noted that only TCD4^+^ cells express GcgR. The percentage of TCD4^+^ cells that expressed GcgR was 7.0 ± 1.0% (n = 6). Meanwhile, the percentage of TCD8^+^cells from mediastinal lymph nodes that expressed GcgR was considered insignificant (data not shown).

**Figure 3:**
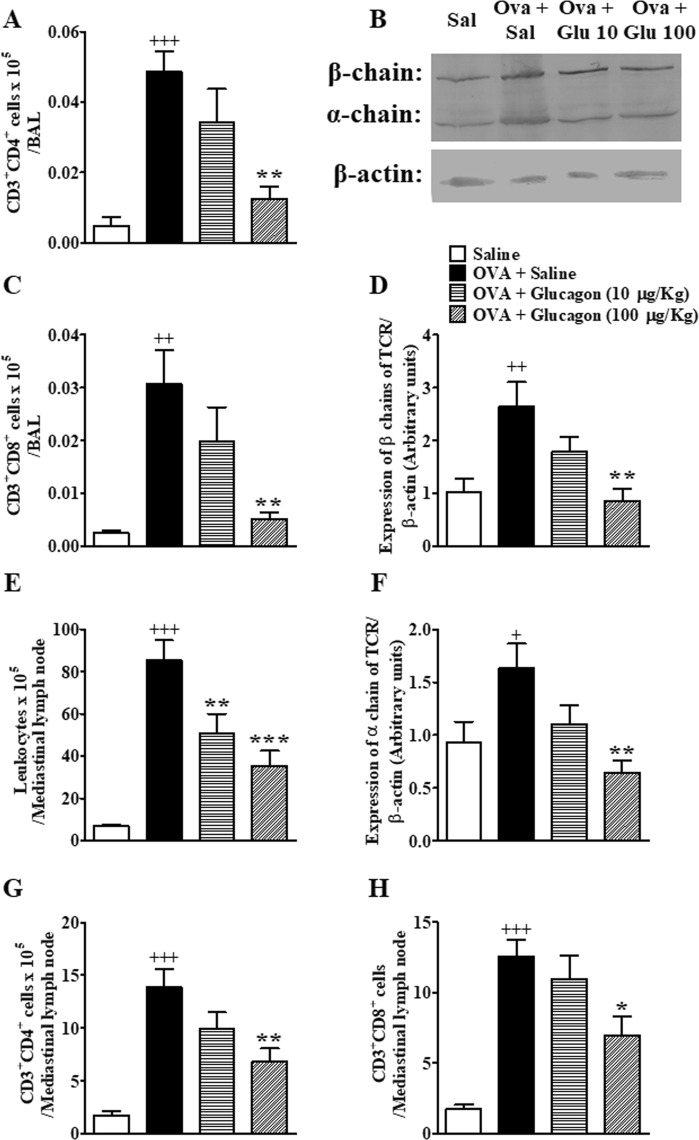
Glucagon prevents elevation of TCD4^+^ and TCD8^+^ in BAL and mediastinal lymph node and inhibits the increase of TCRαβ expression in lungs of A/J mice challenged with OVA. Treatment with glucagon (10 and 100 µg/Kg, i.n.) or its vehicle (sterile saline 0.9%, i.n.) was performed 1 h before each challenge with OVA, once a day, for 2 consecutive days. Numbers of TCD4^+^ and TCD8^+^ cells in BAL (**A**,**C**, respectively) and total leukocytes, TCD4^+^ and TCD8^+^ cells in mediastinal lymph node (**E**,**G**,**H**, respectively). (**B**) Representative images of the expression of TCRαβ determined by western blot. Densitometry analysis of the expression of β (**D**) and α chains (**F**) of TCR in the lungs of A/J mice challenged i.n. with OVA and treated with glucagon or sterile saline. Full-length blots of TCR α and β chain and β-actin are reported in Supplementary Fig. S5.The results are expressed as the mean ± SEM of 4–6 animals per group. Statistical analysis was performed using one-way ANOVA followed by Newman–Keuls-Student’s T test. ^+^*P* < 0.05 compared to the group challenged with saline. ^++^*P* < 0.01 compared to the group challenged with saline. ^+++^*P* < 0.001 compared to the group challenged with saline. *P < 0.05 compared to the group challenge with OVA plus saline. **P < 0.01 compared to the group challenge with OVA plus saline. ***P < 0.001 compared to the group challenge with OVA plus saline. SAL = Saline. OVA = Ovalbumin. GLU = Glucagon.

Furthermore, allergen challenge increased TCD4^+^ and TCD8^+^ cells in BAL (Fig. 3A,C, respectively), as well as the expression of α and β chains of TCR in lung tissue samples (Fig. 3D,F, respectively) compared to sham-challenged mice. Only the dose of 100 μg/Kg of glucagon i.n. prevented OVA-induced elevation in the numbers of TCD4^+^ and TCD8^+^ lymphocytes in BAL fluid (Fig. 3A,C, respectively), and in the expression of both the α and β chains of the TCR in lung tissue samples (Fig. 3D,F, respectively). Nevertheless, glucagon (10 µg/Kg, i.n.) had no significant effect in the increase of TCD4^+^ and TCD8^+^ lymphocytes (Fig. 3A,C, respectively) in addition to TCR α and β expression (Fig. 3D,F, respectively) induced by OVA in BAL and lung tissue samples, respectively. Representative images of α and β chains of TCR expression are shown in Fig. 3B. Full-length blots of TCR α and β chain and β-actin are reported in Supplementary Fig. S5.

In mediastinal lymph nodes, OVA challenge induced an increase in the number of total leukocytes compared to mice challenged with saline. Both doses of glucagon (10 and 100 µg/Kg) inhibited the increase of total leukocytes induced by OVA in mediastinal lymph nodes (Fig. 3E). This OVA-induced elevation of total leukocytes recovered from mediastinal lymph nodes was accompanied by a rise in the numbers of TCD4^+^ and TCD8^+^ lymphocytes (Fig. 3G,H, respectively). Glucagon at 100 μg/Kg inhibited TCD4^+^ (Fig. 3G) and TCD8^+^ (Fig. 3H) cell changes in mediastinal lymph nodes, remaining inactive at the dose of 10 μg/Kg (Fig. 3G,H, respectively).

### Glucagon prevents OVA-induced increase in Eotaxin-1/CCL11, Eotaxin-2/CCL24, IL-4, IL-5, IL-13, and TNF-α, but not macrophage-derived chemokine/CCL22 and thymus-activation-regulated chemokine/CCL17 in the lungs

**Figure 4:**
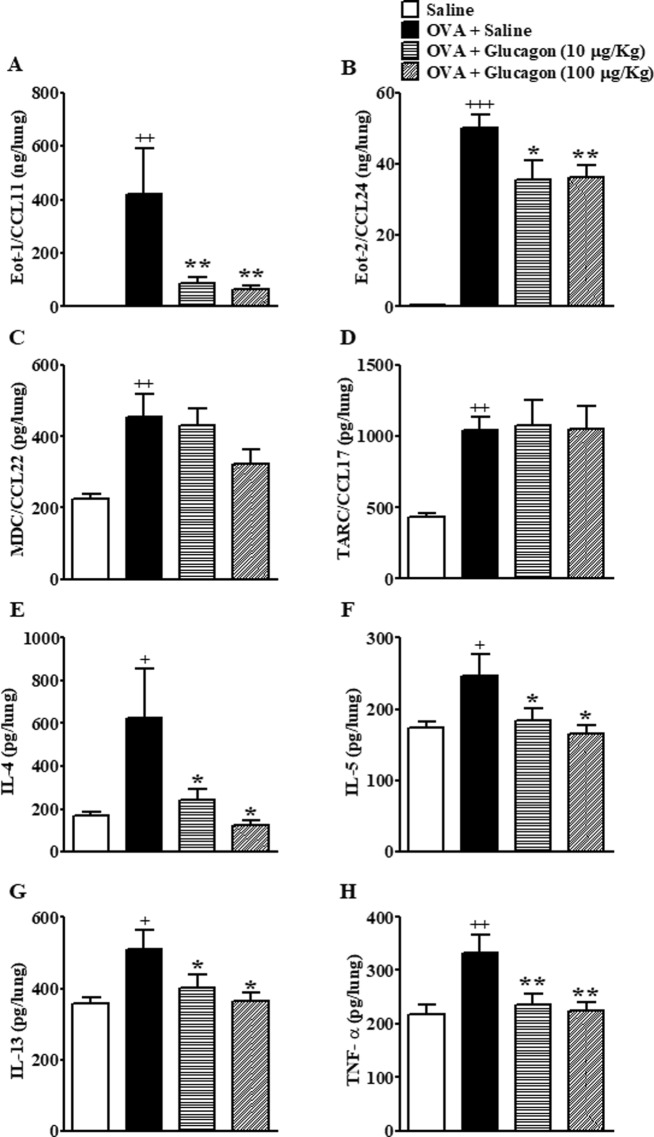
Glucagon prevents chemokines and cytokine generation in the lung tissue of A/J mice challenged with OVA. The treatment with glucagon (10 and 100 μg/Kg, i.n.) or its vehicle (sterile saline 0.9%, i.n.) was performed 1 h before each challenge with OVA, once a day, for 2 consecutive days. Lung tissue levels of Eot-1/CCL11 (**A**), Eot-2/CCL24 (**B**), MDC/CCL22 (**C**), TARC/CCL17 (**D**), IL-4 (**E**), IL-5 (**F**), IL-13 (**G**), and TNF-α (**H**) were evaluated 24 hours after the last i.n. challenge with OVA (25 μg/25 μL) or saline. The levels of the chemokines and cytokines were quantified by ELISA. The results are expressed as the mean ± SEM of 8–10 animals per group. Statistical analysis was performed using a one-way ANOVA followed by Newman–Keuls-Student’s T test. ^+^*P* < 0.05 compared to the group challenged with saline. ^++^*P* < 0.01 compared to the group challenged with saline. ^+++^*P* < 0.001 compared to the group challenged with saline. *P < 0.05 compared to the group challenge with OVA plus saline. **P < 0.01 compared to the group challenge with OVA plus saline.

The challenge with OVA increased Eotaxin- (Eot)-1/CCL11, Eot-2/CCL24, macrophage-derived chemokine (MDC)/CCL22, thymus-activation-regulated chemokine (TARC)/CCL17, IL-4, IL-5, IL-13, and TNF-α levels in lungs compared to mice challenged with saline (Fig. 4). Glucagon (10 and 100 µg/Kg, i.n.) prevented the rise in Eot-1/CCL11 (Fig. 4A), Eot-2/CCL24 (Fig. 4B), IL-4 (Fig. 4E), IL-5 (Fig. 4F), IL-13(Fig. 4G), and TNF-α (Fig. 4H) without inhibiting production of MDC/CCL22 (Fig. 4C) and TARC/CCL17 (Fig. 4D).

### Glucagon impairs allergen-induced remodeling in lung tissue

**Figure 5:**
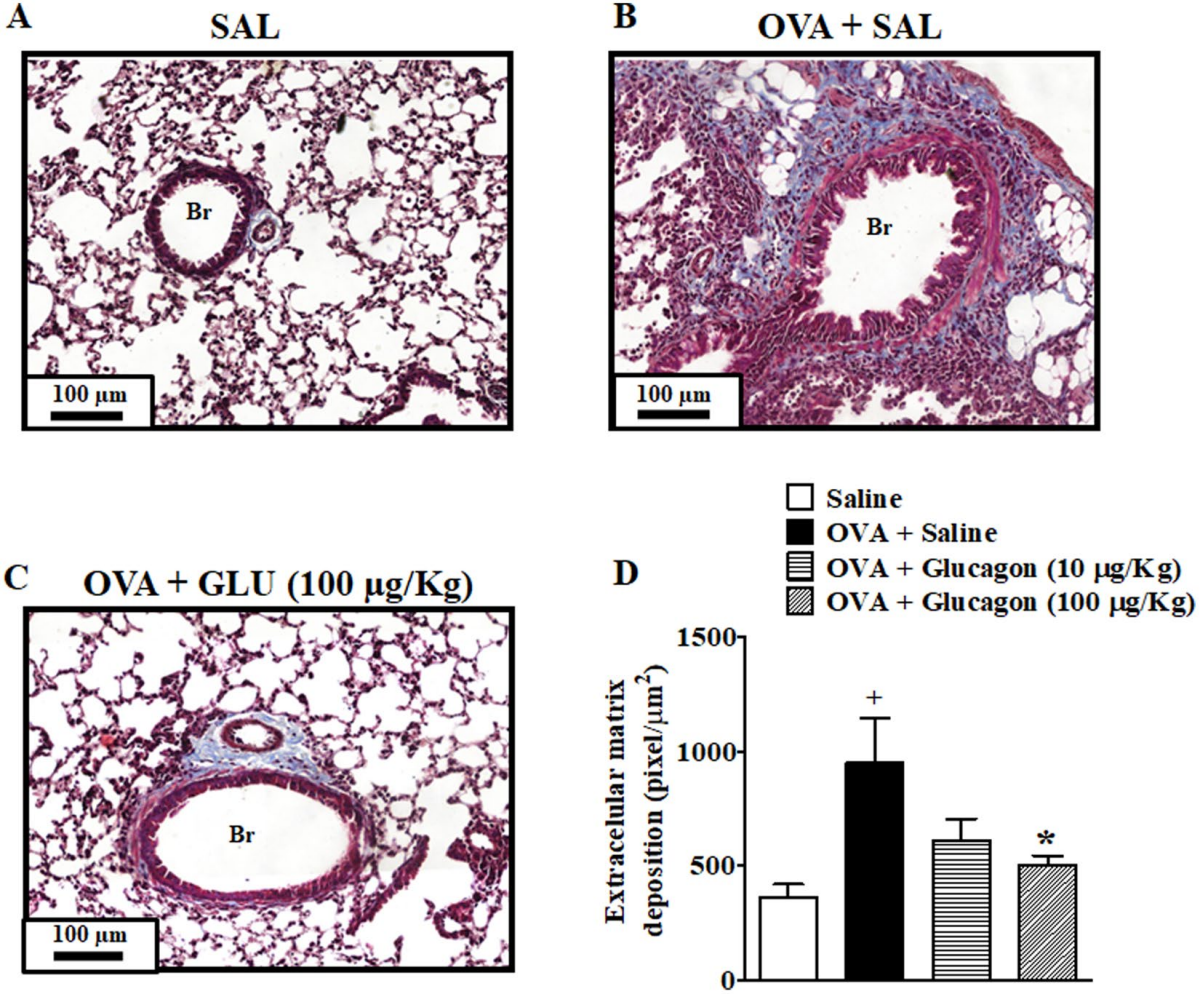
Glucagon prevents subepithelial peribronchiolar fibrosis induced by OVA challenge. The treatment with glucagon (10 and 100 μg/Kg, i.n.) or its vehicle (sterile saline 0.9%, i.n.) was performed 1 h before each challenge with OVA, once a day, for 2 consecutive days. Photomicrographs of representative lung histologic sections stained Masson’s Trichrome from saline-challenged (0.9%, i.n.) (**A**), OVA-challenged mice (25 µg/25 µL, i.n.) treated with sterile saline (0.9%, i.n.) (**B**), and OVA-challenged mice treated with glucagon 100 µg/Kg, i.n. (**C**). Subepithelial peribronchiolar fibrosis (**D**) was quantified digitally in 8–10 airways per animals. The results are expressed as the mean ± SEM of 5 animals per group. Statistical analysis was performed using a one-way ANOVA followed by Newman–Keuls-Student’s T test. ^+^*P* < 0.05 compared to the group challenged with saline. *P < 0.05 compared to the group challenge with OVA plus saline. Br = Bronchiolar lumen. SAL = Saline. OVA = Ovalbumin. GLU = Glucagon.

To access the putative effect of glucagon on allergen-induced lung remodeling, we have quantified peribronchiolar deposits of the extracellular matrix in sensitized and allergen challenged mice subjected or not to intranasal glucagon treatment. Our findings show that allergen provocation significantly increased the deposit of extracellular matrix (Fig. 5B) compared to sham-challenged mice (Fig. 5A) which was clearly sensitive to glucagon (100 µg/Kg) (Fig. 5C). Quantitative data are shown in Fig. 5D.

### Inhibition of OVA-induced AHR and remodeling by glucagon depends of COX products

**Figure 6:**
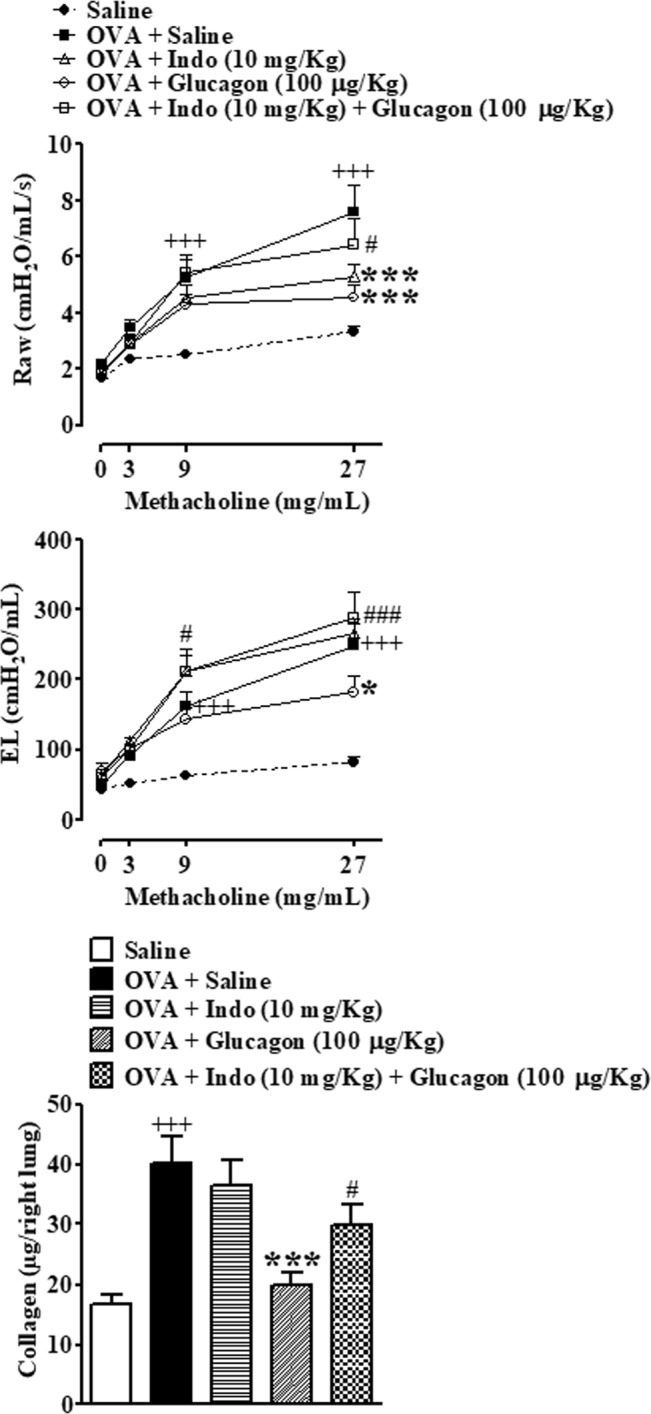
Indomethacin prevented the inhibitory effect of glucagon on OVA-induced AHR to methacholine and collagen deposition in the lungs of A/J mice. Indomethacin (10 mg/Kg, i.p.) abolished the protective effect of glucagon (100 µg/Kg, i.n.) on increase of Raw (**A**), EL (**B**) and lung collagen content (**C**) in A/J mice challenged i.n. with sterile saline (0.9%) or OVA (25 μg/25 μL). The treatment with glucagon (100 μg/Kg, i.n.) or its vehicle (sterile saline 0.9%, i.n.) was performed 1 h before each challenge with OVA, once a day, for 2 consecutive days. Indomethacin or its vehicle (DMSO 0.3%) was injected 30 min before each glucagon treatment. The results are expressed as the mean ± SEM of 6–8 animals per group. Statistical analysis was performed using a two-way ANOVA followed by Bonferroni post-test for AHR evaluation. Statistical analysis was performed by ANOVA followed by Newman–Keuls-Student’s T test for collagen evaluation. ^+++^*P* < 0.001 compared to the group challenged with saline. *P < 0.05 compared to the group challenge with OVA plus saline. ***P < 0.001 compared to the group challenge with OVA plus saline. ^#^*P* < 0.05 compared to the group challenge with OVA and treated with glucagon 100 µg/Kg. ^###^*P* < 0.001 compared to the group challenge with OVA and treated with glucagon 100 µg/Kg. OVA = Ovalbumin. Indo = Indomethacin.

To investigate the possible mechanism involved in the ability of glucagon in inhibits OVA-induced AHR and tissue remodeling, a non-selective COX inhibitor indomethacin (10 mg/Kg) was injected intraperitoneally 30 min before glucagon (100 µg/Kg). Glucagon treatment clearly inhibited OVA-induced AHR regarding both Raw (Fig. 6A) as well as EL changes (Fig. 6B), and indomethacin abrogated the glucagon protective effect concerning methacholine-induced changes in Raw and EL (Fig. 6A,B). Indomethacin did not alter OVA-induced increase in EL (Fig. 6B), but surprisingly inhibited improved in Raw evoked by OVA (Fig. 6A). As expect, challenge with OVA increased collagen deposition in the lungs compared to animals provoked with saline, and treatment with glucagon prevented OVA-induced collagen deposition. Pre-treatment with indomethacin blocked the inhibitory properties of glucagon on collagen deposition. Indomethacin did not affect collagen deposition when was injected in untreated mice challenged with OVA (Fig. 6C).

### Allergen increases TCD4^+^ and TCD8^+^ GcgR^+^ populations in BAL and mediastinal lymph nodes

**Figure 7:**
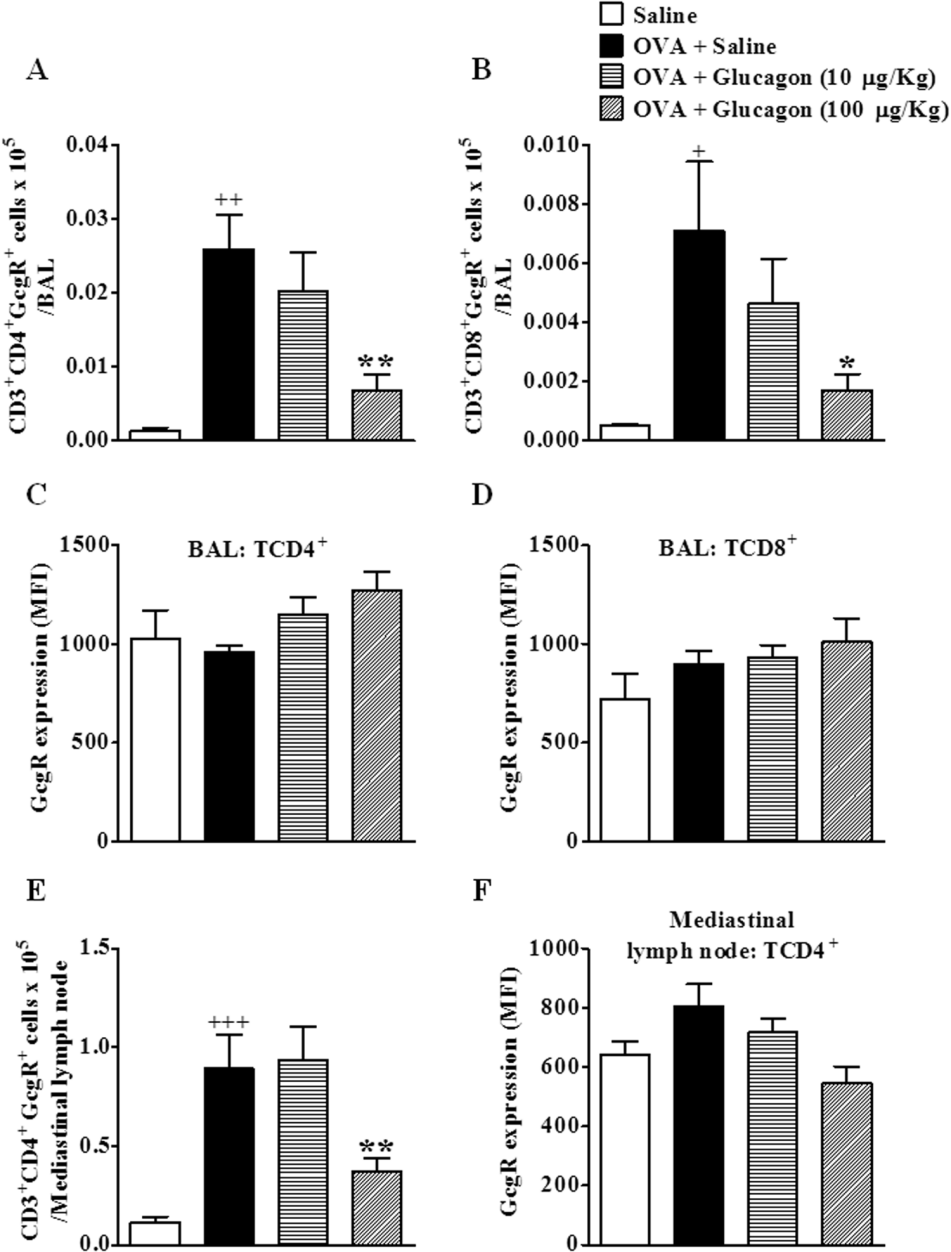
Glucagon inhibits the increase on TCD4^+^ GcgR^+^ and TCD8^+^ GcgR^+^ cells induced by OVA in BAL and mediastinal lymph node. Treatment with glucagon (10 and 100 µg/Kg, i.n.) or its vehicle (sterile saline 0.9%, i.n.) was performed 1 h before each challenge with OVA, once a day, for 2 consecutive days. Numbers of TCD4^+^ GcgR^+^ and TCD8^+^ GcgR^+^ cells in BAL (**A**,**B**, respectively) and TCD4^+^ GcgR^+^ in mediastinal lymph node (**E**). Median fluorescent intensity (MFI) of GcgR on TCD4^+^ (**C**,**F**) and TCD8^+^ (**D**) cells in BAL (**C**,**D**), and on TCD4^+^ cells in mediastinal lymph node (**F**). Isotype control of GcgR in flow cytometry showed less than 3% of positive events in all experiments. The results are expressed as the mean ± SEM of 4–6 animals per group. Statistical analysis was performed using a one-way ANOVA followed by Newman–Keuls-Student’s T test. ^+^*P* < 0.05 compared to the group challenged with saline. ^++^*P* < 0.01 compared to the group challenged with saline. ^+++^*P* < 0.001 compared to the group challenged with saline. *P < 0.05 compared to the group challenge with OVA plus saline. **P < 0.01 compared to the group challenge with OVA plus saline.

We observed that the challenge with OVA induced an increase in the numbers of TCD4^+^ and TCD8^+^ cells that express GcgR in BAL (Fig. 7A,B, respectively), and an elevation in the numbers of TCD4^+^ cells that express GcgR in mediastinal lymph nodes (Fig. 7E) compared to saline-challenged mice. Intranasal instillation of glucagon at 100 µg/Kg, but not at 10 µg/Kg, reduced the numbers of TCD4^+^GcgR^+^ and TCD8^+^GcgR^+^ in BAL (Fig. 7A,B, respectively), and the numbers of TCD4^+^GcgR^+^ in mediastinal lymph node (Fig. 7E). Nevertheless, we did not noted alteration in the median fluorescent intensity (MFI) for GcgR on TCD4^+^ and TCD8^+^ in both BAL (Fig. 7C,D, respectively) and mediastinal lymph node (Fig. 7F) of any group analyzed. The isotype control of GcgR in flow cytometry showed less than 3% of positive events in all experiments and experimental groups.

### Glucagon inhibits proliferation and activation of T lymphocytes stimulated with anti-CD3 or antigen *in vitro*

Based on the effects of glucagon in the murine model of acute asthma induced by OVA *in vivo*, which pointed T lymphocytes as possible targets for anti-asthmatic effects of glucagon, we investigated the direct action of glucagon over the activation of T lymphocytes *in vitro*. For this, we use two protocols for T cell stimulation *in vitro*. First, cells obtained from cervical, axillary and inguinal lymph nodes were treated with dexamethasone (1 μM) or glucagon (0.03–3 μM) and simultaneously stimulated with immobilized anti-CD3 (1 μg/mL) *in vitro* for 72 h. Anti-CD3 promoted an increase in the proliferation of T lymphocytes which was sensitive to 1 μM dexamethasone. Glucagon at concentrations of 0.3 and 3 μM was also able to inhibit anti-CD3-induced T cell proliferation *in vitro* (Fig. S6A). Furthermore, anti-CD3-induced T cell activation up-regulated IL-2, IL-10, and IL-17 production *in vitro* (Fig. S6B–D, respectively). Treatments with either 1 μM dexamethasone or 3 μM glucagon inhibited these responses whereas lower concentrations of glucagon (0.03 and 0.3 μM) inhibited only IL-10 production (Fig. S6B–D).

In the second protocol, the cells were obtained from a pool of cervical, axillary and inguinal lymph nodes of transgenic mice DO11.10 (TCR Tg) and then treated with dexamethasone or glucagon and simultaneously stimulated with soluble OVA (0.5 mg/mL) for 72 h. OVA increased the proliferative response of T lymphocytes (Fig. S6E) as well as IL-13 production (Fig. S6F). Dexamethasone (1 μM) and glucagon (1 and 3 μM) equally inhibited OVA-induced T cell proliferation (Fig. S6E) and IL-13 production (Fig. S6F).

### Glucagon inhibits a combination of anti-CD3 and anti-CD28-induced proliferation and activation of TCD4^+^ cells, and increases intracellular cAMP levels *in vitro*

**Figure 8:**
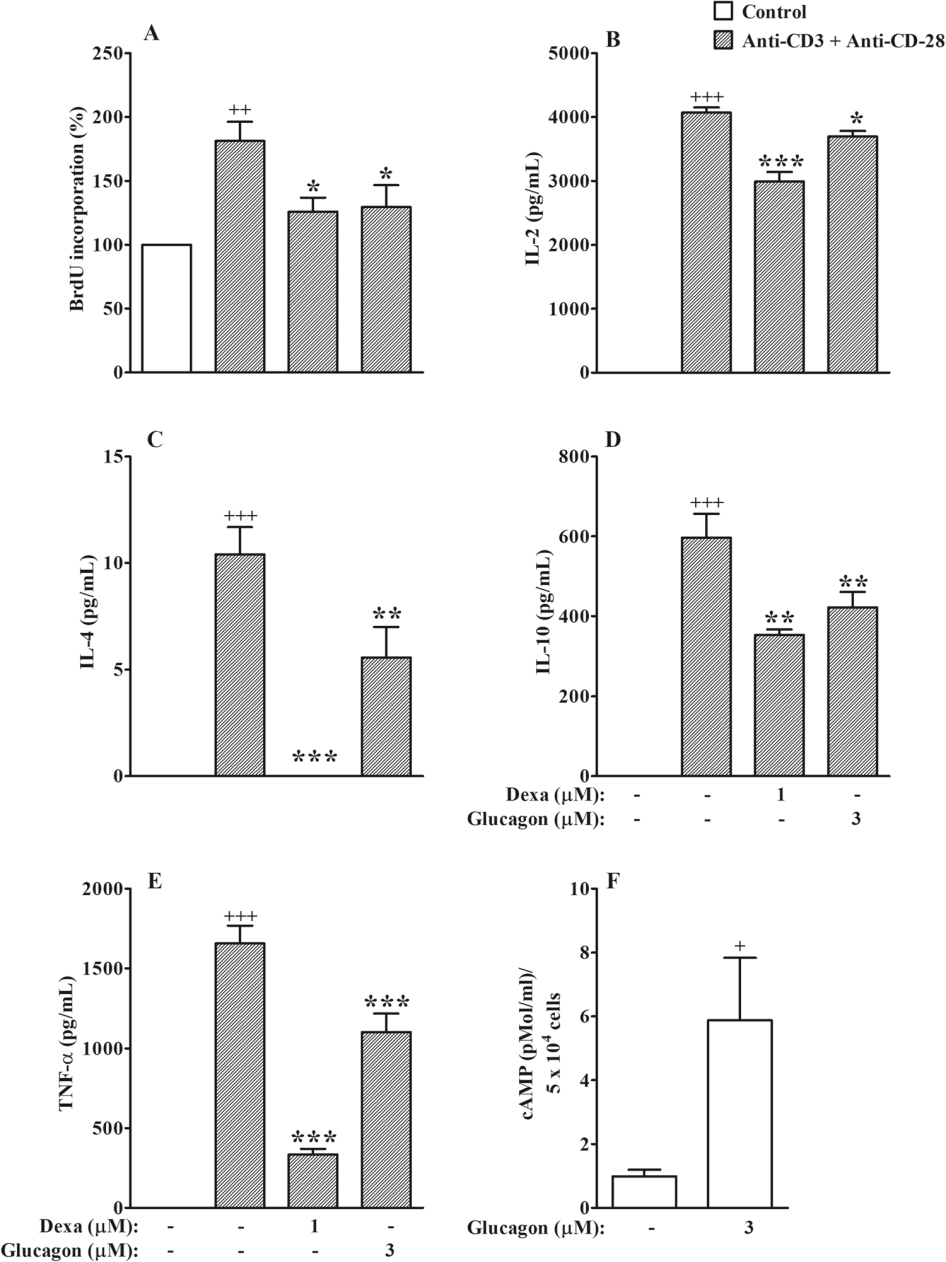
Glucagon increases intracellular cAMP levels and inhibits the proliferative response and cytokine production, by TCD4^+^ cells stimulated *in vitro*. Effect of glucagon on proliferation (**A**) and secretion of IL-2 (**B**), IL-4 (**C**), IL-10 (**D**), and TNF-α (**E**) by TCD4^+^ cells and stimulated with anti-CD3 plus anti-CD28 *in vitro*. Effect of glucagon on the intracellular levels of cAMP in TCD4^+^ cells (**F**). The results are expressed as the mean ± SEM of 4 animals per group. Statistical analysis was performed using a one-way ANOVA followed by Newman–Keuls-Student’s T test. ^+^*P* < 0.05 compared to cells stimulated with sterile saline *in vitro*. ^++^*P* < 0.01 compared to cells stimulated with sterile saline *in vitro*. ^+++^*P* < 0.001 compared to cells stimulated with sterile saline *in vitro*. *P < 0.05 compared to cells stimulated with anti-CD3 plus anti-CD28 *in vitro*. **P < 0.01 compared to cells stimulated with anti-CD3 plus anti-CD28 *in vitro*. ***P < 0.001 compared to cells stimulated with anti-CD3 plus anti-CD28 *in vitro*. Dexa = Dexamethasone.

Since the TCD4^+^ cells are the major T cells involved in the pathogenesis of asthma, and as we observed that glucagon inhibited activation of T lymphocytes *in vitro*, we investigated the direct action of glucagon on TCD4^+^ cells. To confirm the anti-proliferative effect of glucagon on TCD4^+^ cells, we isolated these cells and treated with dexamethasone (1 μM) or glucagon (3 μM) and simultaneously stimulated with immobilized anti-CD3 plus anti-CD-28 *in vitro* for 72 h. Anti-CD3 plus anti-CD28 promoted an increase in the proliferation of TCD4^+^ cells which was sensitive to 1 μM dexamethasone. Glucagon was also able to inhibit anti-CD3 plus anti-CD28-induced TCD4^+^ cell proliferation *in vitro* (Fig. 8A). Then, we evaluated the ability of glucagon in inhibit cytokine production by TCD4^+^ cells *in vitro*. We showed that treatments with either 1 μM dexamethasone or 3 μM glucagon significantly inhibited anti-CD3 plus anti-CD28-induced TCD4^+^ cell activation up-regulated IL-2, IL-4, IL-10, and TNF-α production *in vitro* (Fig. 8B–E, respectively). Finally, we noted that glucagon induced an increase in the intracellular levels of cAMP (Fig. 8F), with values similar to that observed when we stimulated TCD4^+^ cells with forskolin, an adenylyl cyclase activator (4.4 ± 1.1 cAMP (pMol/ml)/5 × 10^4^ cells, n = 4, mean ± SEM).

## Discussion

In the present study, we showed that the i.n. instillation of glucagon prevented major pathological changes caused by allergen challenge in actively sensitized mice, including AHR, lung infiltration of eosinophils, TCD4^+^ and TCD8^+^ cells, expansion of TCD4^+^ and TCD8^+^ cells in the mediastinal lymph node as well as increase in pro-inflammatory mediators in association with significant reduction of subepithelial fibrosis. Furthermore, we observed that sensitized mice challenged with OVA exhibited an increase in the number of inflammatory cells expressing GcgR in peribronchial areas, including mononuclear and polymorphonuclear cells, mediastinal lymph nodes, and BAL effluent, such as total CD3^+^ cells, TCD4^+^ cells, dendritic cells, and neutrophils, but we did not shown modifications in the number of eosinophils which express GcgR. Since all these inflammatory cells express GcgR, our data indicate that those seem to be crucial cell targets in asthma pathophysiology which are modulable by glucagon. Glucagon treatment decreased the number of cells recovered in the BAL which express GcgR, which can be explained by the fact that glucagon can promote desensitization of its own receptor^16^. The possibility does exist that auto-desensitization might explain the down-regulation of the GcgR. However, glucagon did not alter the frequency of cells GcgR^+^ in mediastinal lymph nodes. Surprisingly, glucagon at 10 µg/Kg increased GcgR expression in the lugs of OVA challenged mice. This data was unexpected and needs further studies to understand this phenomenon. It is noteworthy that a marked up-regulation in the expression of glucocorticoid receptors has also been identified in asthma conditions^17^ with glucocorticoids being, by far, the most effective class of anti-inflammatory drugs to treat asthma.

Smooth muscle is considered the master effector cell in airway bronchoconstriction and hyperreactivity in asthma^18,19^. We found that glucagon prevented an OVA-induced AHR following exposure to methacholine at doses of 10 and 100 µg/Kg but not at 0.1 and 1 µg/Kg, which is in line with previous findings describing the bronchodilator action of glucagon in asthmatic patients^19,20^. Moreover, glucagon inhibited airway smooth muscle contraction caused by cholinergic stimulus *in vitro* and *in vivo* settings by a mechanism involving production of nitric oxide and prostaglandin E_2_ (PGE_2_)^20^. In fact, we showed that inhibition of PGE2 synthesis using indomethacin abrogated the protective effect of glucagon on OVA-induced AHR in mice. On the other hand, since airway inflammation is deeply implicated in the state of AHR in asthmatics^21^, the possibility does exist that a putative anti-inflammatory action of glucagon might also play a role in this context. Indeed, we showed that glucagon inhibits eosinophil accumulation triggered by OVA in the BAL and lungs, without altering the infiltration of mononuclear cells. Eosinophils are pivotal effector cells in the pathophysiology of asthma. They act via release of several inflammatory mediators, causing lung tissue damage and perpetuate the inflammatory response^17,22^. In most asthmatics, there is a positive correlation between the severity of AHR and the number of eosinophils in the lungs^23^, leading to the interpretation that inhibition of OVA-triggered AHR induced by glucagon may, at least in part, be accounted for by reduction in the eosinophil accumulation in BAL and lungs. Furthermore, AHR can also be associated with the action of some pro-inflammatory cytokines, including IL-13 and TNF-α. Exogenous IL-13 promoted AHR whereas mice deficient in IL-13 and injection of anti-IL-13 monoclonal antibodies in wild type mice reduced AHR after OVA challenge^11,24^. TNF-α can act directly on smooth muscle and increase the contractile response to several spasmodic agents which can contribute to AHR in asthma. Indeed, it was described that the blockade of TNF-α reduced AHR in patients with moderate or severe asthma^10,14^. In our work, glucagon reduced both IL-13 and TNF-α level in the lungs of mice challenged with OVA, which may have also contributed to the inhibitory effect of glucagon on AHR. We believe that the reduced of OVA-induced AHR induced by glucagon depends of anti-inflammatory effects of glucagon and not by a direct action on airway epithelial or smooth muscle cells, once we did not observed immunolabel to GcgR receptors in these cells after OVA challenge.

In parallel to inhibition of eosinophil infiltration, we showed that glucagon reduces the levels of eotaxin-1/CCL11, eotaxin-2/CCL24, IL-4, IL-5, IL-13, and TNF-α in the lungs of mice challenged with OVA. A decrease in the levels of the most of these pro-inflammatory mediators can explain the reason why glucagon inhibited the accumulation of eosinophils, once eotaxin-1/CCL11 and eotaxin-2/CCL24 are chemoattractant for eosinophils^25^; IL-4 induces activation of eosinophils and expression of adhesion molecules by endothelial cells including VCAM-1, which is important for eosinophil extravasation and trafficking^26^; IL-5 promotes the differentiation of eosinophils in the bone marrow and prolongs the survival of eosinophils in the airways; IL-13 has been implicated in eosinophil survival, activation, and recruitment^27^.

Another important hallmark in asthma is the airway remodeling. It is accounted for by thickening of the airway epithelium layer and collagen deposition and strongly contributes to the severity of this disease^25,28^. Our findings showed that glucagon prevents subepithelial fibrosis in mice challenged with OVA. We previously reported that glucagon increases PGE_2_ levels in the lungs^29^. This is relevant since it is well established that PGE_2_ decreases proliferation, collagen production and differentiation in myofibroblasts of pulmonary fibroblasts^30^. Therefore, the up-regulation on PGE_2_ levels might also contribute to the inhibitory effect of glucagon on allergen-induced subepithelial fibrosis in our conditions. In this work, we noted that indomethacin, a non-selective COX inhibitor, partially decreased the protective effect of glucagon on OVA-induced lung collagen deposition in mice. In addition, it is known that IL-13 and TNF-α have an important role in lung fibrosis^14,31^. The reduction of IL-13 and TNF-α level in the lungs after glucagon treatment can also cooperate with the inhibitory action of glucagon on subepithelial fibrosis induced by OVA challenge in mice.

Bearing in mind that also TCD4^+^ lymphocytes play an important role in asthma pathogenesis, coordinating and participating in the onset and progression of the inflammatory response^32^, we evaluated the effect of glucagon on the accumulation of TCRαβ lymphocytes in the lungs, BAL, and mediastinal lymph nodes of mice challenged with OVA. In fact, we showed that glucagon prevented an OVA-induced increase of numbers of TCD4^+^ and TCD8^+^ cells in BAL and mediastinal lymph nodes and inhibited the elevation of TCRαβ expression in lungs. The reduction in TCRαβ expression indicates that glucagon also inhibits T cell accumulation in lungs triggered by OVA^33^. However, glucagon inhibited OVA-induced T cells numbers in BAL, lungs and mediastinal lymph nodes only at dose of 100 µg/Kg, contrary to what we observe in the parameters of AHR, eosinophil accumulation in BAL and lungs, and eotaxin-1, eotaxin-2, IL-4, IL-5, IL-13, and TNF-α amount in the lungs, in which glucagon was effective at both doses used. Together, these data suggest that T cells are not the master inflammatory cell modulated by glucagon in our model of allergen-induced lung inflammation, but probably eosinophils.

Since glucagon did not inhibit the production of chemokines implicated in chemotaxis of Th2 lymphocytes, such as MDC/CCL22 and TARC/CCL17^34^, we evaluated the putative impact upon the expression of GcgR by these cells. We demonstrated that allergen challenge clearly increased the number of TCD4^+^ and TCD8^+^ cells expressing GcgR in BAL, and TCD4^+^ cells expressing GcgR in lymph node, indicating that glucagon could act on activation, proliferation and polarization of these cells. In fact, glucagon treatment decreased the numbers of TCD4^+^ and TCD8^+^ cells expressing GcgR.

While investigating whether glucagon can affect activation and/or proliferation of T lymphocytes, two distinct approaches were employed. First, we stimulated cells obtained from pooled cervical, axial and inguinal lymph nodes from naïve mice with anti-CD3 *in vitro*. Treatment with glucagon inhibited the proliferation of T cells in a concentration-dependent manner. We also showed that glucagon inhibited, in a concentration-dependent manner, the secretion of IL-2, IL-10, and IL-17 by anti-CD3-activated T cells, suggesting that glucagon can indeed inhibit the activation of T lymphocytes. Similar findings were demonstrated when we obtained T cells from lymph nodes of transgenic mice DO11.10 with TCR specific for OVA, and then activated these cells with OVA *in vitro*. Under this condition we found that glucagon inhibited the proliferation of T cells and the generation of IL-13 caused by allergen challenge. It is noteworthy that glucagon inhibited proliferation and activation of T lymphocytes induced by either anti-CD3 or OVA without altering the viability of these cells, suggesting that this inhibitory action occurred without glucagon inducing cytotoxicity. Furthermore, we noted that glucagon also inhibited proliferation and activation, attested by production of IL-2, IL-4, IL-10, and TNF-α, of TCD4^+^ cells induced by a combination of anti-CD3 and anti-CD28 *in vitro*. The reduction in the proliferation and activation of T lymphocytes, especially TCD4^+^ cells, by glucagon *in vitro* may help to explain the reduction in the number of TCD4^+^ and TCD8^+^ cells in mediastinal lymph node and BAL and TCRαβ expression in lungs of OVA-challenged mice treated with glucagon. It has been reported that an increase in intracellular levels of cAMP inhibits proliferation, IL-2 production and functional activity of T cells. In addition, cAMP also increases the expression of CTLA-4 and induces anergy in T cells at rest^35^.

The modulation of cAMP intracellular levels has been considered an important therapeutic target for asthma^36^. This is because elevations in intracellular levels of cAMP inhibit several elements of the inflammatory response and the functions of several structural cells into the airways and lungs. Remarkably, cAMP is a second messenger that activates multiple cell signaling pathways for several proteins and peptides that act controlling homeostasis, including glucagon^37^. The actions of glucagon in the organism occur due to the activation of its receptor, GcgR, which is highly expressed in the liver and kidneys, but is also found in lower levels in many other organs, including trachea and lungs^8,14,38^. GcgR is a member of the Gs protein-bound receptor family and thus the activation of this receptor by of glucagon stimulates adenylate cyclase with subsequent increase in intracellular levels of cAMP^39^. cAMP exerts its effects mainly through the stimulation of cAMP-protein kinase A (PKA), which phosphorylates several molecules, including the cAMP response element-binding protein (CREB)^40,41^. Here, we showed that glucagon increased the intracellular levels of cAMP in TCD4^+^ cells with same amount that noted when we stimulated TCD4^+^ cells with an adenylyl cyclase activator. Thus, we believe that the inhibitory effect of glucagon on anti-CD3 plus anti-CD28-stimulated TCD4^+^ lymphocytes *in vitro* can be occurring through GcgR, since it has already been described to play a role both in receptor expression of TCD4^+^ cells and in an increase in cAMP produced by glucagon in these cells^42,43^.

Although it could be expected that the main adverse effect of glucagon therapy would be hyperglycemia, actually the central side effect of the administration of this hormone is nausea and vomiting^44^. In fact, the parenteral injection of glucagon in asthmatic patients is generally well tolerated, with patients commonly reported only nausea as adverse reaction^11,45^. However, when glucagon was given locally in the airways by nebulization, similar to what we performed, in asthmatic patients, no adverse effects was observed^10,46^. In addition, the major shortcoming of the use of glucagon for asthma treatment is that some authors showed that glucagon alone is ineffective for the management of asthma exacerbations^47^, despite its ability to induce bronchodilation, and that several authors found that glucagon was effective for treatment of asthmatic patients, including in the chronic phase of the disease^10,11,45,46,48^.

In conclusion, our findings demonstrate that glucagon reduces the AHR, inflammatory response, and remodeling induced by allergen in a murine model of acute asthma. The bronchodilator and anti-remodeling effects of glucagon seem to be at least partially dependent of induction of PGE2 in the lungs. This anti-inflammatory action appears to be accounted for by a decrease in recruitment of eosinophils and T lymphocytes to the airways and by blockade of TCD4^+^ cell proliferation. These data give support to the possibility of exploiting glucagon as a therapeutic agent in asthma control.

## Materials and Methods

### Chemicals

Dexamethasone, glucagon, indomethacin, methacholine, nembutal, and OVA (Grade V) were purchased from Sigma Chemical Co (St. Louis, USA). Pancuronium bromide and sodium thiopental were purchased from Cristália (São Paulo, Brazil). Dexamethasone, glucagon, OVA, pancuronium bromide, nembutal, and sodium thiopental were dissolved in 0.9% NaCl sterile solution. Methacholine was diluted in phosphate-buffered saline (PBS). Indomethacin was prepared in DMSO 0.3%. All of the solutions were freshly prepared immediately before use.

### Animals

Six to eight-week-old (18–20 g) male A/J mice and DO11.10 TCR Tg mice were obtained from the Oswaldo Cruz Foundation breeding colony and used in accordance with the guidelines of the Committee on Use of Laboratory Animals of the Oswaldo Cruz Institute (CEUA-IOC/FIOCRUZ), and all the experimental protocols used in this work were approved by CEUA-IOC/FIOCRUZ (license L-027/2016). Mice were housed in groups of five in a temperature, humidity, and light controlled (12 h light/dark period) colony room. Mice were given *ad libitum* access to food and water.

### Sensitization, antigen challenge, and treatment protocol

A/J mice were sensitized with a subcutaneous injection of sterile saline solution (0.2 mL) containing OVA (50 µg) and the adjuvant aluminum hydroxide Al(OH)_3_ (5 mg). After 14 days, all mice were boosted following the same procedure. On the 19^th^ day after sensitization, the animals were subjected to an i.n. challenge of OVA (25 μg/25 μL) or sterile saline (0.9%) for 2 consecutive days, once daily. Analyses were performed 24 h after the last challenge using OVA, which corresponded to the 21^st^ day after sensitization. Treatment with glucagon (0.1–100 µg/Kg, i.n.) or its vehicle (sterile saline 0.9%, i.n.) were performed once a day 1 h before each OVA challenge. In some experiments, the animals were pretreated intraperitoneally with indomethacin (10 mg/Kg, i.p.)^14^ or its vehicle (DMSO 0.3%) 30 min before glucagon injection (100 µg/Kg, i.n.).

### Bronchoalveolar Lavage

Twenty-four hours after the last challenge using sterile saline or OVA, A/J mice were euthanized by injection of sodium thiopental (500 mg/Kg, i.p.). Then, the airways were lavaged with two injections of 0.75 mL of PBS containing 10 mM methylenediaminetetraacetic acid (EDTA). BAL fluid was centrifuged (300 × g, 10 min, 4 °C) and cell pellets were resuspended in 250 μL of PBS plus EDTA (10 mM). To quantify the leukocyte influx into the airway lumen, BAL effluent was diluted in Türk solution (2% acetic acid) and total leukocytes were counted in a Neubauer chamber, using a light microscope (BX40; Olympus, Center Valley, PA). Differential cell counts were performed in cytospin smears stained with the May-Grünwald Giemsa method and analyzed using a light microscope (BX40, Olympus).

### Analysis of mediastinal lymph node cells

After the last challenge using sterile saline or OVA, the mediastinal lymph node was extracted, mechanically macerated and cell aggregates were removed using a plastic strainer (Cell strainer 40 μm, BD Biosciences Pharmingen, San Jose, CA, USA) in a petri dish with Dulbecco’s modified Eagle’s medium (DMEM; Sigma-Aldrich) supplemented with penicillin 1 × 10^6^ U/Ml (Sigma-Aldrich); 0.2 g/mL streptomycin (Sigma-Aldrich); 10% fetal bovine serum (FBS). The macerate was centrifuged (433 x g for 10 min at 4 °C), and then, the pellet was resuspended in 1 mL of the supplemented DMEM. All samples were diluted in the Turk dye solution (2% acetic acid) for the quantification of total cells in a Neubauer chamber using a light microscope (BX40; Olympus).

### Immunohistochemistry

Paraffin-embedded sections (5 µM) of the left lung were boiled in 10 mM sodium citrate (pH 6.0) for 15 min for antigen retrieval. To block endogenous peroxidase activity, the tissue sections were incubated with hydrogen peroxide (3%) in methanol for 20 min. Non-specific binding sites were blocked using FBS (8%), bovine serum albumin (BSA, 2.5%), and non-fat milk (1%) diluted in Tris-buffered saline (TBS) enriched with 0.1% Tween 20 (TBST) for 3 h. All sections were incubated overnight at 4 °C with primary polyclonal rabbit anti-GcgR (1:100; Santa Cruz Biotechnology, Santa Cruz, USA) antibody diluted in TBST plus 1% BSA. Then, we incubated the sections with horseradish peroxidase-conjugated streptavidin (HRP) (polyclonal anti-rabbit IgG (1:100), R&D System, MN, USA) for 2 h 30 min at room temperature followed by a 20-min exposure to the enzyme substrate 3-amino-9-ethylcarbazole (AEC). The sections were counterstained using hematoxylin for 10 s to visualize the structure of the lungs. In negative control experiments, the primary antibody was omitted. The slides were scanned using a 3DHISTECH–Pannoramic MIDI whole slide scanner (Budapest, Hungary) and captured with a 20× objective lens. The analyses were performed using Image-Pro-Plus^®^ software version 6.2 (Media Cybernetics Inc, Bethesda, MA, USA), and the number of positive pixels was divided by the field area and expressed as pixels/*μ*m^2^.

### Invasive assessment of airway hyperreactivity to methacholine

AHR was determined as an increase reactivity of the airways to aerosolization of methacholine 24 h after the last sterile saline or OVA challenge. Mice were anesthetized using nembutal (60 mg/Kg, i.p.), and neuromuscular activity was blocked by pancuronium bromide (1 mg/Kg, i.p.). Pulmonary function and AHR were assessed in tracheostomized and mechanically ventilated mice using a FinePointe R/C Buxco Platform (Buxco® Electronics, Sharon, CT, USA), as previously described^14^.

### Eosinophil peroxidase activity in lung tissue

The EPO activity assay is a well-established and accurate method for measuring the number of eosinophils in biological samples, as described previously^16,17^. This method allows quantification of the reaction of peroxidases secreted by eosinophils through an optical absorbance measurement^18^. Briefly, one lobe of the right lung was homogenized in 5% Hank’s Balanced Salt Solution (HBSS) and centrifuged at 960 × *g* for 10 min at 4 °C. After red blood cells lysis, we added 5% HBSS containing 0.5% hexadecyltrimethyl ammonium bromide to samples and performed three successive cycles of freezing and thawing. After centrifugation (1090 × *g* for 10 min at 4 °C), the samples were placed (75 μL/well) in a 96-well plate, and then, we added 150 μL of substrate (1.5 mM *o*-phenylenediamine and 6.6 mM hydrogen peroxide in 0.05 M Tris-HCl, pH 8.0) and incubated for 30 min at room temperature. Then, the reaction was stopped by the addition of 75 μL of 4 M sulfuric acid and the absorbance was read at 492 nm (Spectra Max M5, Molecular Devices, Sunny vale, CA, USA). The results are represented as optical density (OD) per ρg of protein in each sample. Protein was quantified by the Bicinchoninic acid (BCA) assay (Sigma-Aldrich Corp).

### Evaluation of GcgR expression on T cells, TCD4^+^ cells, TCD8^+^ cells, eosinophils, neutrophils, and dendritic cells

Cells obtained from BAL or mediastinal lymph nodes of mice challenged with OVA or sterile saline *in vivo* were analyzed by flow cytometry (FACSAria II; BD Biosciences PharMingen or FACSCalibur; BD Biosciences PharMingen). We labeled the cells using monoclonal antibodies anti-CD3 (FITC or PECF-594), anti-CD4 (APC or FITC), anti-CD8 (PECy5), anti-SiglecF (PE) (BD Biosciences PharMingen), anti-Ly6G (FITC), anti-CD11c (PE) (Thermo Fisher Scientific, Swedesboro, NJ, USA) or polyclonal antibody anti-GcgR (PE) (Bioss Antibodies, Woburn, MA, USA). To evaluated GcgR expression on Total CD3^+^ cells, TCD4^+^ cells, eosinophils, neutrophils, and dendritic cells in BAL, we incubated these cells with primary polyclonal rabbit anti-GcgR (Santa Cruz Biotechnology) antibody for 30 min, washed with PBS, and then we incubated the cells with polyclonal anti-rabbit-Alexa 635 antibody (BD Biosciences PharMingen) for 30 min. In control nonspecific binding, we used an isotype-matched antibodies or the primary polyclonal rabbit antibody anti-GcgR (Santa Cruz Biotechnology) was omitted. The results were analyzed using Summit software version 4.3 (Beckman Coulter.). Gating strategies for identification of populations are shown in Figs S2, S3, S7 and S8.

### Western blot

One lobe of the right lung was homogenized in cold lysis buffer containing a cocktail for protease and phosphatase inhibitors (1:100; Thermo Fisher Scientific). The homogenates were centrifuged (13,000 × g for 10 min at 4 °C), the supernatant was collected, and the total proteins were quantified by using BCA method (Sigma–Aldrich Corp). Total proteins (50 µg/lane) were resolved using 10% SDS-PAGE and then transferred to nitrocellulose membranes. The membranes were blocked using nonfat dry milk (5%) in TBS for 1 h at room temperature and incubated overnight at 4 °C with biotin conjugated anti-TCR α/β (1:100; MyBioSource, San Diego, USA). Next, the membranes were rinsed in blocking buffer and incubated with HRP-secondary antibody (Dako, Carpinteria, CA) for 1 h at room temperature. For loading control, nitrocellulose membranes were incubated with anti-β-actin (1:5000; Sigma-Aldrich Corp.) overnight at 4 °C, rinsed in blocking buffer, and incubated with HRP goat anti-mouse (1:10,000; R&D Systems) for 1 h at room temperature. Detections were performed using 3,3′-diaminobenzidine (DAB)-peroxidase substrate solution (Vector Labs, Burlingame, CA). The membranes were scanned, and band intensity was quantified by densitometry using the software Image Studio Lite version 4.0 (LI-COR Corporate, Lincoln, NE, USA).

### Quantification of cytokines and chemokines in lungs

One lobe of the right lung was homogenized in cold lysis buffer containing the protease inhibitor cocktail Complete (F. Hoffmann-La Roche Ltd., Basel, Switzerland) and 0.1% Triton X-100 in PBS 1X. After centrifugation (13,000 × *g* for 10 min at 4 °C) of the lysate, the supernatant levels of murine Eot-1/CCL11, Eot-2/CCL24, MDC/CCL22, and TARC/CCL17, IL-4, IL-5, IL-13, and TNF-α were measured using a commercially available enzyme linked immunosorbent assay (ELISA) kits (R&D Systems) according to the manufacturer’s instructions. The results were expressed as ρg of cytokine or chemokine per lung.

### Measurement of Pulmonary Inflammation and Peribronchiolar Fibrosis

The left lung was fixed in Milloning buffer solution (pH 7.4) with 4% paraformaldehyde to preserve the pulmonary architecture. Lung sections were stained using Sirius Red (pH 10.2) or Masson’s trichrome for the analysis of peribronchiolar leukocyte infiltration and extracellular matrix deposits, respectively, as described previously^19,20^. The slides stained using Masson’s trichrome were scanned with 3DHISTECH–Pannoramic MIDI whole slide scanner, and the images were captured using a 20× objective lens. The analysis was performed in 10 bronchioles per animal in a double-blind experiment evaluating the use of Image-Pro-Plus^®^ software version 6.2 (Media Cybernetics Inc,). The quantification of eosinophils, neutrophils and mononuclear cells number on the peribronchiolar region was performed under an optical microscope (BX40; Olympus) through an integrating eyepiece. The morphometric reticulum had a known area of 10^4^ μm^2^ at the final magnification of 1,000× and was randomly positioned over the peribronchiolar regions. Three different fields were analyzed for bronchioles in 7 bronchioles per animal^19,20^.

### Quantification of lung collagen

The right lung was homogenized in Tris-HCl 0.05 M and NaCl 1 M solution containing Complete (Hoffmann-La Roche) (pH 7.4). Total soluble collagen was extracted overnight at room temperature and its levels were measured using Sircol kit (Biocolor, Newton Abbey, U.K.) according to the manufacturer’s instructions. The results were expressed as milligrams collagen per right lung^27^.

### Analysis of T cell proliferation and cytokine production *in vitro*

Pooled T lymphocytes from cervical, axial and inguinal lymph nodes (1 × 10^6^/well) were obtained from A/J mice or naïve DO11.10 TCR Tg mice and stimulated with immobilized anti-CD3 (1 µg/mL; BD Biosciences Pharmingen) or soluble OVA (0.5 mg/mL), respectively. Cells were incubated with dexamethasone (1 μM) or glucagon (0.03–3 μM) for 72 h at 37 °C in 5% CO_2_. For the analysis of cell viability, aliquots of cell suspensions were mixed with trypan blue solution (0.2%) and all cells presented at least 85% of viability in all groups studied. Proliferation was analyzed by permeabilizing and staining the cells with propidium iodide (PI) as previously described^21^. Proliferation values were determined by calculating the percentage of cells in phase S + G2 of the cell cycle. In the protocol of T cells stimulated with anti-CD3 *in vitro*, cytokines IL-2, IL-10 and IL-17 were detected from the supernatant of these cells using a Cytometric Bead Array (CBA) kit (BD Biosciences PharMingen), while in OVA-induced T cells activation *in vitro*, the cytokine IL-13 was evaluated by ELISA kits (R&D Systems), according to the manufacturer’s instructions.

### Isolation of mice naïve TCD4^+^ cells

Pooled cells from cervical, axial and inguinal lymph nodes were obtained from A/J naïve mice and TCD4^+^ cells were isolated by magnetic bead depletion of TCD8^+^ cells, B cells, monocytes/macrophages, NK cells, dendritic cells, erythrocytes, and granulocytes (Dynal Mouse CD4 negative Isolation Kit, Invitrogen, Carlsbad, CA, USA). After isolation, the purity of TCD4^+^ cells, attested by flow cytometry (FACSCalibur; BD Biosciences PharMingen), was higher than 93%. Gating strategies for identification of CD3^+^CD4^+^ population in lymph nodes before or after negative isolation are shown in Fig. S9.

### Analysis of TCD4^+^ cell proliferation and cytokine production *in vitro*

Isolated TCD4^+^ cells (2 × 10^4^/well) were obtained from A/J mice and stimulated with immobilized anti-CD3 plus anti-CD-28 (0.5 µg/ml and 1 µg/mL, respectively; BD Biosciences Pharmingen). Cells were incubated with dexamethasone (1 μM) or glucagon (3 μM) for 72 h at 37 °C in 5% CO_2_. Proliferation was measured by BrdU incorporation using the Cell Proliferation ELISA, BrdU colorimetric kit (Millipore, Burlington, MA, USA) according to the manufacturer’s instructions. To evaluate the production of cytokines IL-2, IL-4, IL-10, and TNF-α, isolated TCD4^+^ cells (5 × 10^5^ cells/well) were stimulated with immobilized anti-CD3 plus anti-CD-28 (1 and 2 µg/mL, respectively) and were detected from the supernatant of these cells using a CBA kit (BD Biosciences PharMingen).

### Intracellular cAMP quantification

For cAMP quantification, 5 × 10^4^ TCD4^+^ cells were treated with 500 nM of IBMX (a competitive nonselective phosphodiesterase inhibitor) and, after 15 min, with glucagon (3 µM) or forskolin (100 µM), for 20 min. Culture supernatants were removed, cells were lysed with 0.1 M HCl, and intracellular cAMP levels were determined by ELISA according to the manufacturer’s instructions (Cayman Chemical, Ann Arbor, MI, USA).

### Statistical Analysis

The data were reported as the mean ± standard error of the mean (SEM) and then evaluated to ensure a normal distribution. All data were statistically analyzed using a one-way ANOVA followed by Newman–Keuls–Student’s t-test except for the study of AHR, which was examined using two-way ANOVA followed by Bonferroni post-test, and the quantification of intracellular cAMP, which was analyzed by unpaired t-test, with GraphPad Prism 5.0 (Graphpad Software, San Diego, CA, USA). Probability values (P) of 0.05 or less were considered significant.

## Supplementary information

Supplementary information

## Data Availability

The datasets generated during and/or analyzed during the current study are available from the corresponding author on reasonable request.
