# Supplementary material for: Glucagon reduces airway hyperreactivity, inflammation, and remodeling induced by ovalbumin
Source: Sci Rep. 2019 Apr 24;9:6478. doi: 10.1038/s41598-019-42981-6 (PMC6482309; doi:10.1038/s41598-019-42981-6)
Supplement: Supplementary file 1 — Supplementary information [file 41598_2019_42981_MOESM1_ESM.docx]

**Supplementary information (online only)**

**Glucagon reduces airway hyperreactivity, inflammation, and remodeling induced by ovalbumin**

Daniella B.R. Insuela^1^, Carolina T.Azevedo^1^, Diego S. Coutinho^1^, Nathalia S. Magalhães^1^, Maximiliano R. Ferrero^1,^ Tatiana Paula T. Ferreira^1^, Cynthia M. Cascabulho^2^, Andrea Henriques-Pons^2^, Priscilla C. Olsen^3^, Bruno L. Diaz^4^; Patricia M.R. Silva^1^, Renato S. B. Cordeiro^1^, Marco A. Martins^1^and Vinicius F. Carvalho^1,4^*.

^1^Laboratory of Inflammation, Oswaldo Cruz Institute, Oswaldo Cruz Foundation (FIOCRUZ), Rio de Janeiro, Brazil; ^2^Laboratory of Innovations in Therapies, Education and Bioproducts, Oswaldo Cruz Institute, Oswaldo Cruz Foundation (FIOCRUZ), Rio de Janeiro, Brazil; ^3^Laboratory of Clinical Bacteriology and Immunology, Department of Toxicological and Clinical Analysis, Faculty of Pharmacy, Federal University of Rio de Janeiro, Rio de Janeiro, Brazil; ^4^Laboratory of Inflammation, Carlos Chagas Filho Institute of Biophysics, Federal University of Rio de Janeiro, Rio de Janeiro, Brazil; ^5^National Institute of Science and Technology on Neuroimmunomodulation (INCT-NIM), Brazil

**
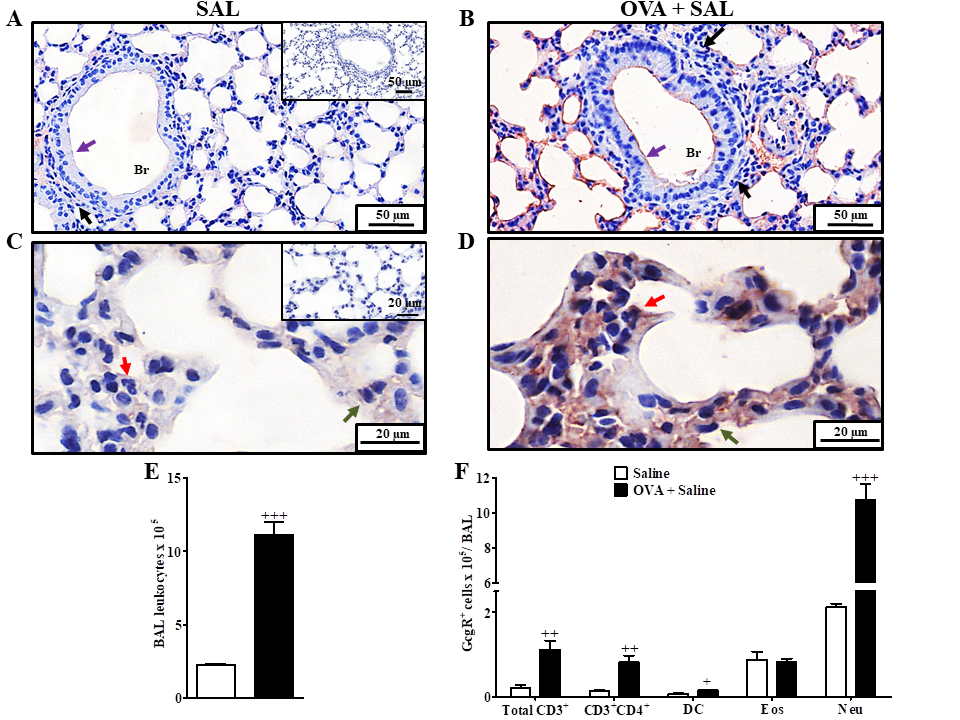
**

**Figure S1. OVA-increased the number of Total CD3^+^ cells (T cells), TCD4^+^cells, dendritic cells, and neutrophils that express GcgR^+^in the BAL and increased the expression of GcgR in mononuclear and polymorphonuclear cells in the lungs of A/J mice.**The animals were challenged i.n. with OVA (25 μg/25 μL) or sterile saline (0.9%) once a day for 2 consecutive days. Lungs were removed and BAL was collected for analysis 24 h after the last challenge. Representative photomicrographs of GcgR expression on bronchioles and peribronchiolar region of lungs of A/J mice provoked with sterile saline (A and C) or OVA plus saline (B and D). Purple arrows indicate epithelial cells. Black arrows indicate smooth muscle cells. Red arrows indicate polymorphonuclear cells. Green arrows indicate mononuclear cells. The inserted figure shows the negative control of immunolabeling. Br = Bronchiolar lumen. SAL = Saline. OVA = Ovalbumin.BAL leucocytes numbers (E) and numbers of Total CD3^+^ cells, TCD4^+^cells, dendritic cells, eosinophils and neutrophils that express GcgR^+^ in the BAL (F). The results are expressed as the mean ± SEM of 3-6 animals per group. Statistical analysis was performed using a one-way ANOVA followed by Newman–Keuls-Student's T test. +*P*<0.05 compared to the group challenged with saline. ++*P*<0.01 compared to the group challenged with saline.+++*P*<0.001 compared to the group challenged with saline. Total CD3^+^ cells = T cells. TCD4^+^ = TCD4^+^ cells. DC = dendritic cells. Eos = Eosinophils. Neu = Neutrophils.

**
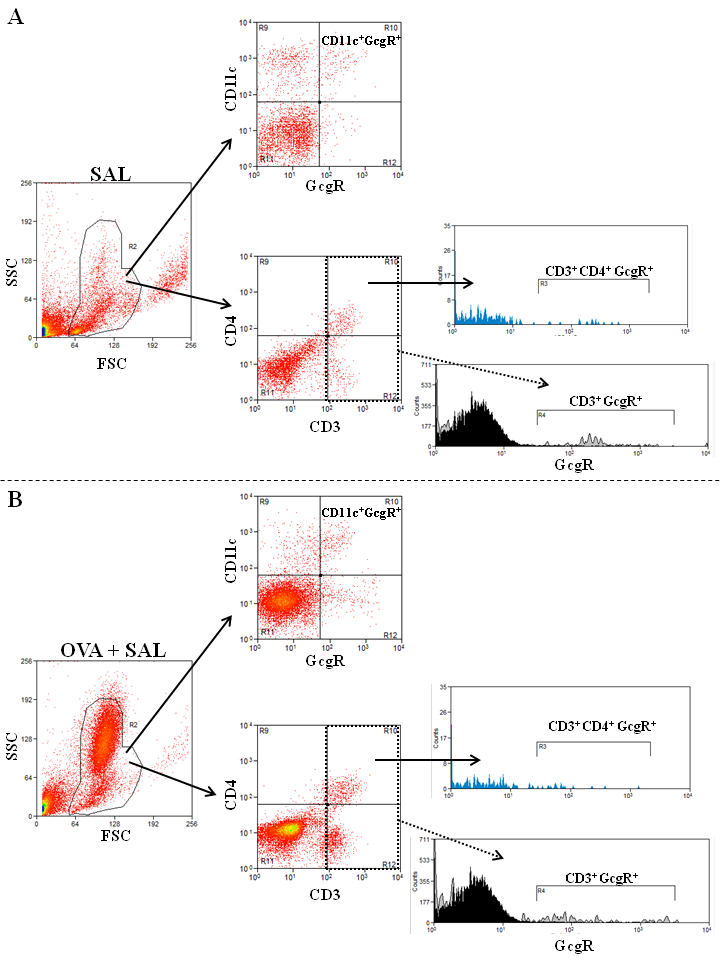
Figure S2. Gating strategies to identify the expression of GcgR on total T, TCD4^+^ and dendritic cells recovered by BAL in all samples of the experiment.** Dot plots and histograms of cells populations from BAL of saline- (0.9%, i.n.) (A) and OVA-challenged mice (25 µg/25 µL, i.n.) treated with sterile saline (0.9 %, i.n.) (B). For identification of T and dendritic cells, BAL samples were stained with monoclonal antibodies anti-CD3 (PECF-594), anti-CD4 (FITC) or anti-CD11c (PE). To evaluated GcgR expression in these cells types we incubated cells with primary polyclonal rabbit anti-GcgR antibody and next with polyclonal anti-rabbit-Alexa 635 antibody. T cells that express GcgR were identified as CD3^+^GcgR^+^; TCD4^+^ cells as CD3^+^CD4^+^GcgR^+^ and dendritic cells as CD11c^+^GcgR^+^. Isotype-matched antibodies were used to control nonspecific binding. Furthermore, to control nonspecific binding of GcgR, some samples were incubated only with anti-rabbit-Alexa 635 and showed less than 3% of positive events in all experiments and experimental groups.


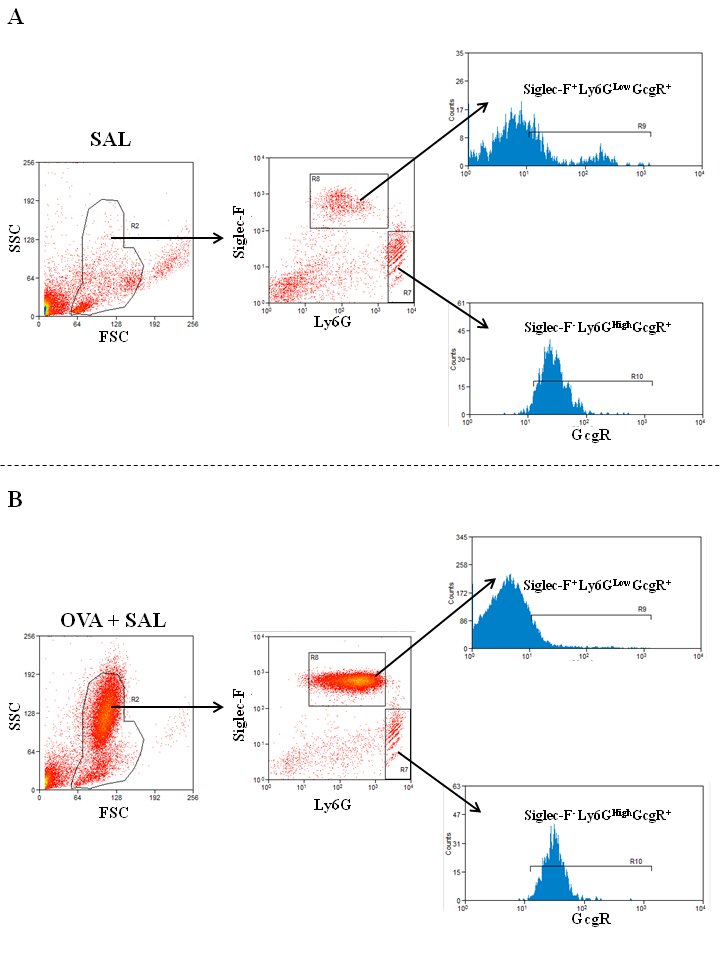
**Figure S3. Gating strategies to identify the expression of GcgR on neutrophils and eosinophils recovered by BAL in all samples of the experiment.** Dot plots and histogram of cells populations from BAL of saline- (0.9%, i.n.) (A) or OVA-challenged (25 µg/25 µL, i.n.) treated with sterile saline (0.9 %, i.n.) groups. For identification of granulocyes types, cells were stained with monoclonal antibodies anti- anti-Siglec-F (PE) and anti-Ly6G (FITC). To evaluated GcgR expression in these cells types we incubated cells with primary polyclonal rabbit anti-GcgR antibody and next with polyclonal anti-rabbit-Alexa 635 antibody. Eosinphils that express GcgR were identified as Siglec-F^+^Ly6G^Low^GcgR^+^; neutrophils as Siglec-F^-^Ly6G^High^GcgR^+^. Isotype-matched antibodies were used to control nonspecific binding. Furthermore, to control nonspecific binding of GcgR, some samples were incubated only with anti-rabbit-Alexa 635 and showed less than 3% of positive events in all experiments and experimental groups.

**
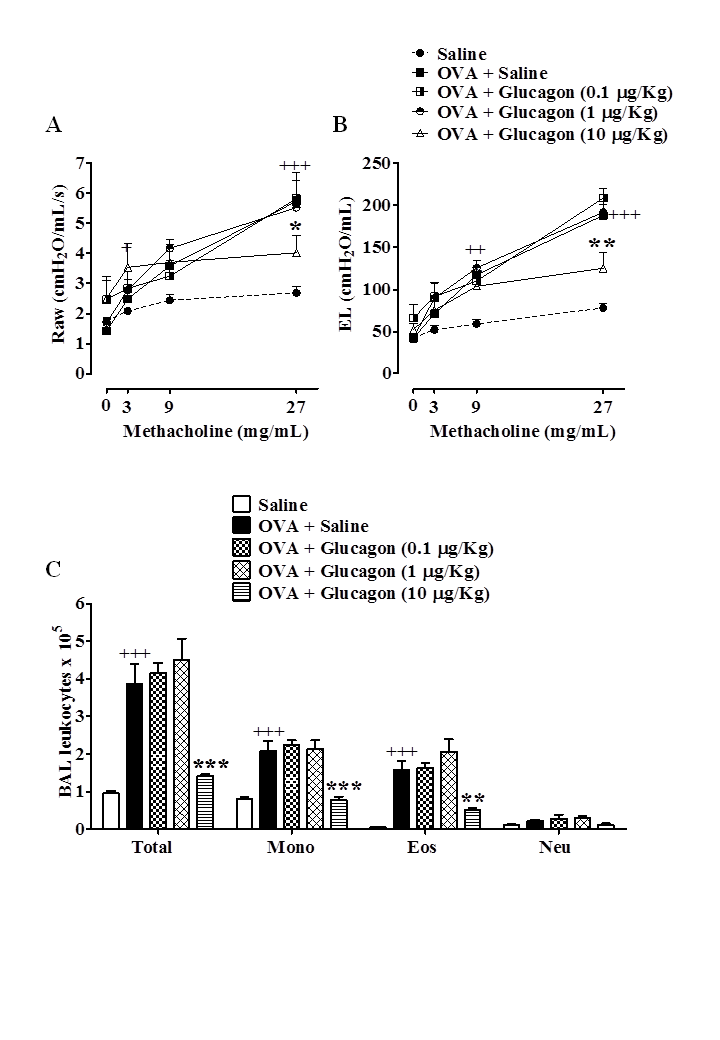
**

**Figure S4. Glucagon inhibits AHR to metacholine and leukocytes accumulation in BALofA/J mice challenged with OVA.** The dose-response analysis of the effect of glucagon (0.1, 1 and 10 µg/Kg, i.n.) on increased RAW (A), EL (B) and BAL leucocytes numbers (C)in A/J mice challenged i.n. with sterile saline (0.9%) or OVA (25 μg/25 μL). The results are expressed as the mean ± SEM of 4-6 animals per group. Statistical analysis was performed using a one-way ANOVA followed by Newman–Keuls-Student's T test. +++*P*<0.001 compared to the group challenged with saline. ***P*<0.01 compared to the group challenge with OVA plus saline. ****P*<0.001 compared to the group challenge with OVA plus saline. Eos = Eosinophils. Mono = Mononuclear. Neu = Neutrophils.

**
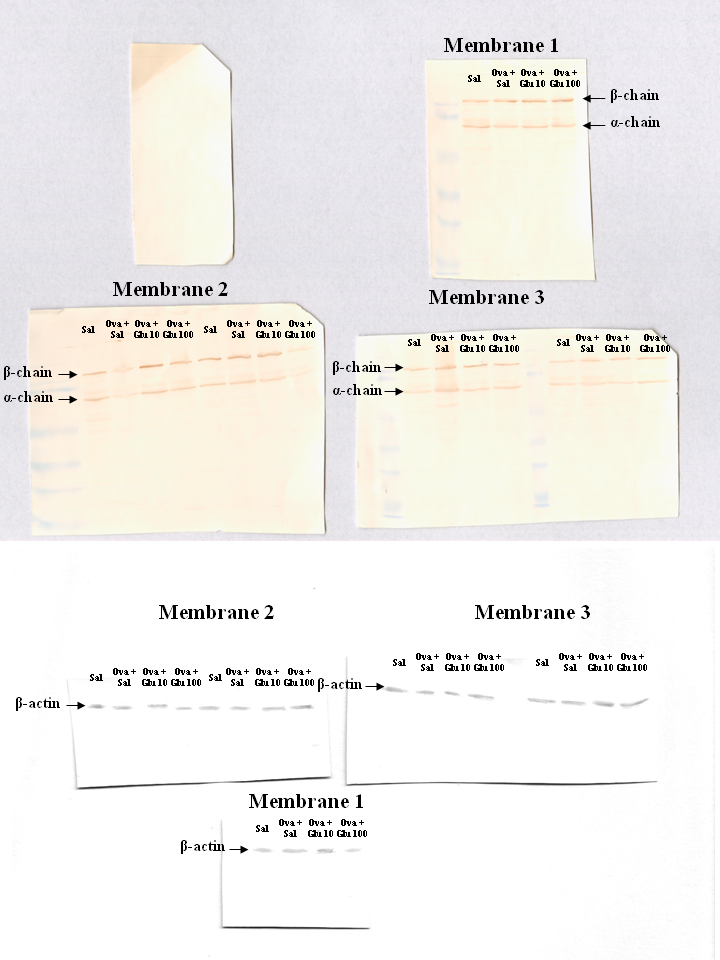
**

**Figure S5. Western blotting evaluation of TCR (β-chain and α-chain) and β-actin expression in A/J mice treated with glucagon (10 and 100 µg/kg, i.n.) or its vehicle (sterile saline 0.9%, i.n.)and challenged with OVA**. Full-length blots of TCR (β-chain and α-chain) and β-actin.

**
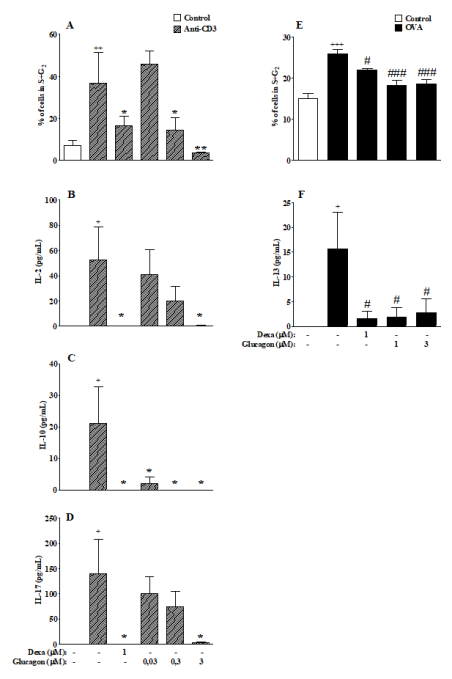
**

**Figure S6. Glucagon inhibits the proliferative response and cytokine production by T cells stimulated *in vitro*.** Effect of glucagon (0.03 – 3 μM) on proliferation (A) and secretion of IL-2 (B), IL-10 (C), and IL-17 (D), by cells recovered from lymph nodes of A/J mice and stimulated with anti-CD3 (1 μg/mL) *in vitro*. Effect of glucagon (1 and 3 μM) on the proliferation of lymphocytes (E) and production of IL-13 (F) by cells recovered from lymph nodes of DO11.10 (TCR Tg) mice and stimulated with OVA (0.5 mg/mL) *in vitro*. The results are expressed as the mean ± SEM of 3-5 animals per group. Statistical analysis was performed using a one-way ANOVA followed by Newman–Keuls-Student's T test. +*P*<0.05 compared to cells stimulated with sterile saline *in vitro*. ++*P*<0.01 compared to cells stimulated with sterile saline *in vitro*. +++*P*<0.001 compared to cells stimulated with sterile saline *in vitro*. **P*<0.05 compared to cells stimulated with anti-CD3 *in vitro*. ***P*<0.01 compared to cells stimulated with anti-CD3 *in vitro*. #*P*<0.05 compared to cells stimulated with OVA *in vitro*. ###*P*<0.001 compared to cells stimulated with OVA *in vitro*. Dexa = Dexamethasone.

**
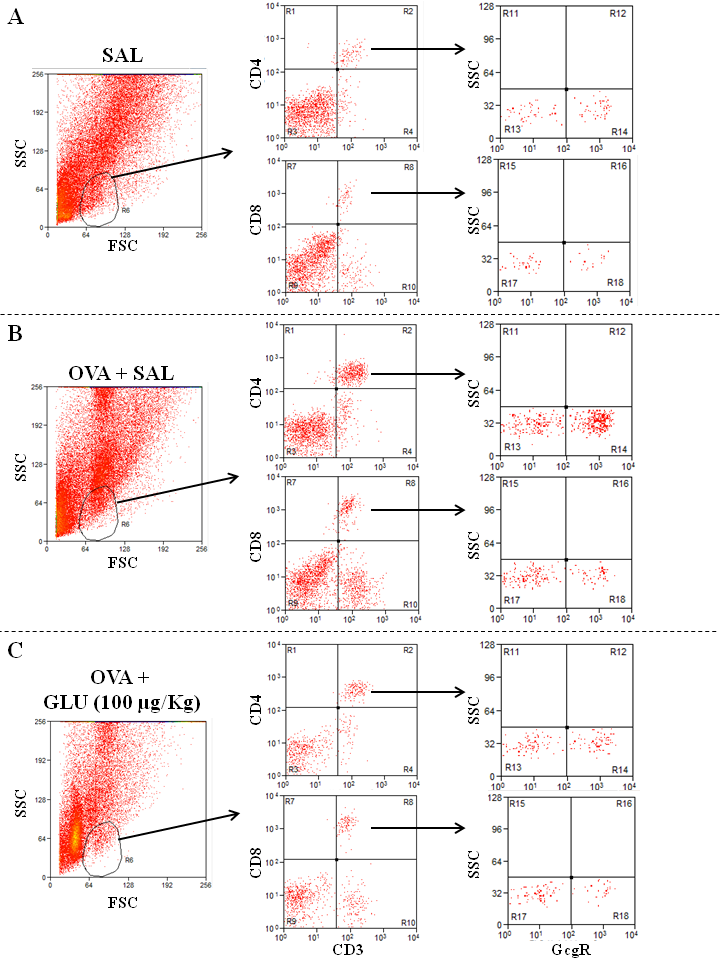
**

**Figure S7. Gating strategies to identify the expression of GcgR on TCD4^+^ cells and TCD8^+^ cellsin BAL.** Cells were stained with monoclonal antibodies anti-CD3-FITC, anti-CD4-APC, anti-CD8-PECy5 or polyclonal antibody anti-GcgR-PE. Dot plots of CD3^+^CD4^+^GcgR^+^ and CD3^+^CD8^+^GcgR^+^populations from saline-challenged (0.9%, i.n.) (A), OVA-challenged mice (25 µg/25 µL, i.n.) treated with sterile saline (0.9 %, i.n.) (B), and OVA-challenged mice treated with glucagon 100 µg/Kg, i.n. (C).The isotype control of GcgR in flow cytometry showed less than 3% of positive events in all experiments and experimental groups.

**
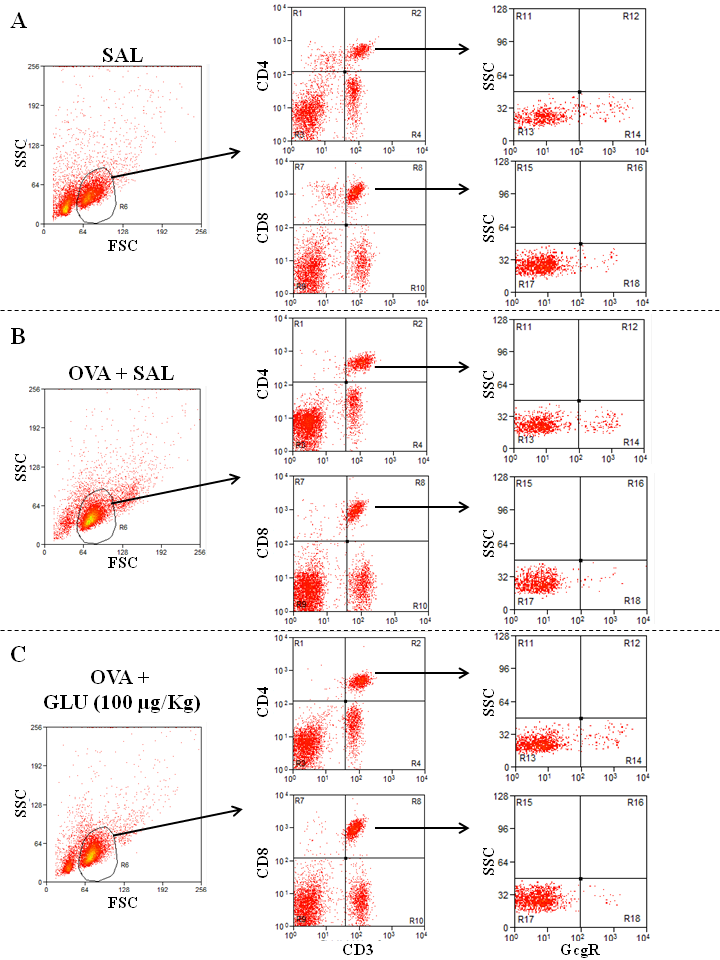
**

**Figure S8.Gating strategies to identify the expression of GcgR on TCD4^+^ cells and TCD8^+^ cells obtained of mediastinal lymph nodes.** Cells were stained with monoclonal antibodies anti-CD3-FITC, anti-CD4-APC, anti-CD8-PECy5 or polyclonal antibody anti-GcgR-PE. Dot plots of CD3^+^CD4^+^GcgR^+^ and CD3^+^CD8^+^GcgR^+^ populations from saline-challenged (0.9%, i.n.) (A),OVA-challenged mice (25 µg/25 µL, i.n.) treated with sterile saline (0.9 %, i.n.) (B), and OVA-challenged mice treated with glucagon 100 µg/Kg, i.n. (C). The isotype control of GcgR in flow cytometry showed less than 3% of positive events in all experiments and experimental groups.

**
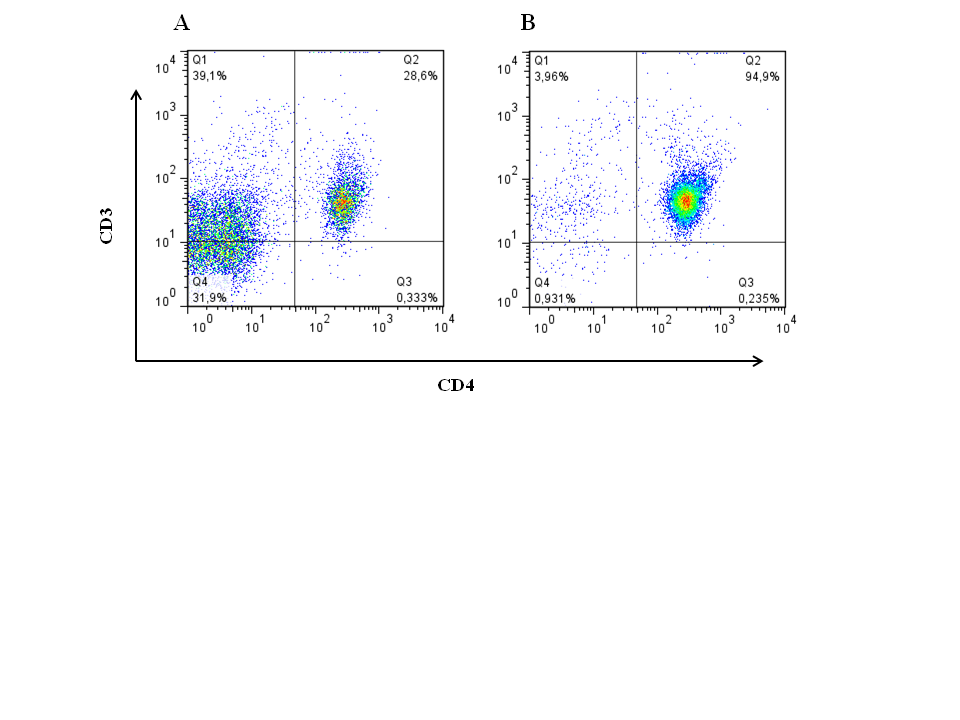
**

**Figure S9. Gating strategies for identification of CD3^+^CD4^+^ population in lymph nodes suspension from A/J mice before (A) or after (B) negative isolation with Dynal Mouse CD4 Negative Isolation Kit.** Cells were stained with monoclonal antibodies anti-CD3-FITC and anti-mouse CD4-APC.
